# Predicting disease severity in metachromatic leukodystrophy using protein activity and a patient phenotype matrix

**DOI:** 10.1186/s13059-023-03001-z

**Published:** 2023-07-21

**Authors:** Marena Trinidad, Xinying Hong, Steven Froelich, Jessica Daiker, James Sacco, Hong Phuc Nguyen, Madelynn Campagna, Dean Suhr, Teryn Suhr, Jonathan H. LeBowitz, Michael H. Gelb, Wyatt T. Clark

**Affiliations:** 1 Translational Genomics Group, BioMarin Pharmaceutical Inc., Novato, CA USA; 2 Department of Pathology and Laboratory Medicine, Children’s Hospital of Philadelphia, Philadelphia, Pennsylvania USA; 3 Department of Pathology and Laboratory Medicine, Perelman School of Medicine, University of Pennsylvania, Philadelphia, Pennsylvania USA; 4 Department of Chemistry, University of Washington, Seattle, WA USA; 5 Department of Biochemistry, University of Washington, Seattle, WA USA; 6 MLD Foundation, West Linn, OR USA

**Keywords:** Metachromatic leukodystrophy, Genotype**–**phenotype relationship, Newborn screening, Mass spectrometry, Phenotype, Mutation

## Abstract

**Background:**

Metachromatic leukodystrophy (MLD) is a lysosomal storage disorder caused by mutations in the arylsulfatase A gene (*ARSA*) and categorized into three subtypes according to age of onset. The functional effect of most *ARSA* mutants remains unknown; better understanding of the genotype–phenotype relationship is required to support newborn screening (NBS) and guide treatment.

**Results:**

We collected a patient data set from the literature that relates disease severity to *ARSA* genotype in 489 individuals with MLD. Patient-based data were used to develop a phenotype matrix that predicts MLD phenotype given *ARSA* alleles in a patient’s genotype with 76% accuracy. We then employed a high-throughput enzyme activity assay using mass spectrometry to explore the function of *ARSA* variants from the curated patient data set and the Genome Aggregation Database (gnomAD). We observed evidence that 36% of variants of unknown significance (VUS) in *ARSA* may be pathogenic. By classifying functional effects for 251 VUS from gnomAD, we reduced the incidence of genotypes of unknown significance (GUS) by over 98.5% in the overall population.

**Conclusions:**

These results provide an additional tool for clinicians to anticipate the disease course in MLD patients, identifying individuals at high risk of severe disease to support treatment access. Our results suggest that more than 1 in 3 VUS in *ARSA* may be pathogenic. We show that combining genetic and biochemical information increases diagnostic yield. Our strategy may apply to other recessive diseases, providing a tool to address the challenge of interpreting VUS within genotype–phenotype relationships and NBS.

**Supplementary Information:**

The online version contains supplementary material available at 10.1186/s13059-023-03001-z.

## Introduction

Metachromatic leukodystrophy (MLD; MIM:250100) is a rare autosomal recessive neurodegenerative disease caused by deficient sulfatide catabolism, due to mutations in the arylsulfatase A gene (*ARSA*) [1]. Patients are generally categorized into three subtypes by age of onset: infantile/late-infantile (onset from 0 up to 2.5 years), juvenile (onset from 2.5 to 16 years), and adult (onset after 16 years) [1]. Upon manifestation of the psychomotor and cognitive symptom characteristic of the disease, prospective MLD patients are diagnosed through neuroradiological, biochemical, and/or genetic testing [1, 2], with enzymatic activity screening being the most common approach [3, 4]. Without treatment, late-infantile and juvenile forms of MLD are fatal and children usually die within years [1, 2]. The efficacy of emergent therapeutics is under investigation, and results suggest that early intervention, in pre-symptomatic patients, is imperative [1, 5–7]. This underscores the importance of newborn screening (NBS) for MLD to support the diagnosis of pre-symptomatic patients.

A pilot study of NBS for MLD used mass spectrometry to measure sulfatide elevation in dried blood spots (first-tier screening), followed by measurement of arylsulfatase A (ARSA) enzyme activity in the same sample (second-tier screening) and *ARSA* genotyping (third-tier screening) [3]. This experiment included over 27,000 newborns and identified two screen positives (one MLD patient true positive, and one MLD carrier false positive), demonstrating the feasibility of NBS for MLD. Screening for MLD would benefit from a more comprehensive understanding of how *ARSA* genotypes affect the severity of MLD and disease prognosis. Genotype–phenotype information for patients in the asymptomatic phase of the disease could guide follow-up and treatment of high-risk patients. It is hypothesized that residual enzymatic activity is inversely proportional to clinical severity.

Although previous work has provided information on MLD genotype–phenotype relationships [8], interpretation of genotypes presents a number of challenges. Variants of unknown significance (VUS) that introduce single amino acid substitutions are often identified, but specific VUS that are rare cannot be assumed to be non-pathogenic [1, 9]. Furthermore, computational methods to accurately predict the effect of single amino acid substitutions on protein function are not sufficient on their own for clinical diagnosis [10]. A second challenge is that not all pathogenic *ARSA* variants are equally damaging to protein function; some are associated with early-onset MLD, whereas others are associated with a later-onset disease [11, 12].

The objective of this study is to develop a predictive model for *ARSA* variants that cause the phenotypic spectrum of MLD. We make progress toward this objective and address the above challenges through a multi-stage process, involving examination of *ARSA* genotypes and MLD phenotypes from the patient literature, collation of *ARSA* variant frequency from the Genome Aggregation Database (gnomAD) and variant disease associations from the ClinVar database, and quantification of ARSA variant enzymatic activities with mass spectrometry [11, 13]. We show that genetic and biochemical information are complementary and that combining the two increases diagnostic yield. We also provide a framework, or phenotype matrix, that explicitly accounts for the impact of each variant in a biallelic individual’s *ARSA* genotype when predicting their MLD phenotype. This framework provides important nuance to the interpretation of variant pathogenicity that is not accounted for in current ACMG guidelines [14], as it allows for genotypes consisting of two pathogenic alleles that may not lead to a recognizable disease phenotype. We make the argument that there are several well-documented autosomal recessive diseases where such pathogenic allele combinations occur, and these scenarios should be carefully considered when conducting newborn screening.

## Results

### The curated patient data set—genotypes and phenotypes

Through a comprehensive literature review, we generated a data set of *ARSA* genotypes and MLD phenotypes for genotype–phenotype analysis; the data set included 489 cases from 49 articles published between 1991 and 2020 (Additional file 1: Table S1). Age of onset data were available for 277 patients, and disease severities/phenotypes were assigned as described in the “Methods.” Three patients were reported without MLD severity or age of onset information and were excluded from our analysis.

**Fig. 1:**
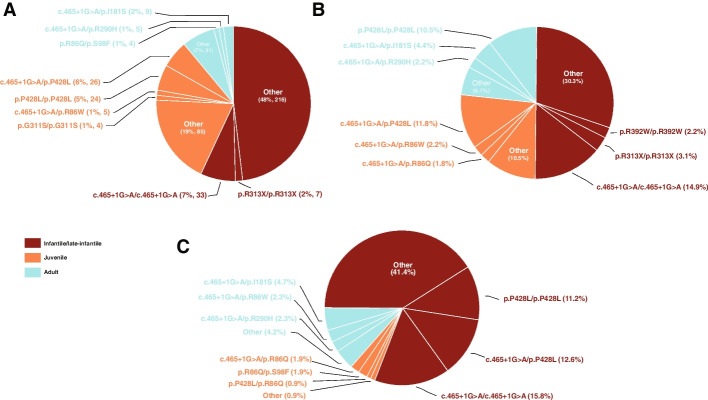
Pie charts of phenotypes and *ARSA* genotypes. **A** Phenotypes in the curated patient data set. The three most common *ARSA* genotypes associated with each severity phenotype are highlighted (*n* = 486). **B** Predicted phenotypes based on the allele frequencies from gnomAD and the patient-based severity ruleset and phenotype matrix. The three most prevalent *ARSA* genotypes for each predicted phenotype are indicated. **C** Predicted phenotypes based on the allele frequencies from gnomAD and ARSA enzyme activities measured in transfected HEK293T cells. The three most prevalent *ARSA* genotypes for each predicted phenotype are indicated. ARSA, arylsulfatase A; gnomAD, Genome Aggregation Database

The distribution of patient phenotypes (*n* = 486) is shown in Fig. 1A. The infantile/late-infantile phenotype of MLD was the most common (*n* = 265, 54.5%), followed by the juvenile phenotype (*n* = 166, 34.2%), with the adult phenotype the least prevalent (*n* = 55, 11.3%). The juvenile category included patients classified in the literature as both “early juvenile” and “late juvenile,” and a patient classified as “mild adult” was annotated as having the adult phenotype in our curation.

A total of 288 distinct genotypes were observed in patients (11 were incomplete, as information was available for only one allele) (Additional file 2: Table S2). c.465 + 1G > A/c.465 + 1G > A was the most frequent, occurring in 33 patients (6.8%); 32 patients were reported as having infantile/late-infantile MLD, while 1 patient was reported with an unknown phenotype. The second most common genotype was c.465 + 1G > A/p.P428L (*n* = 26; 5.3%), with 25 patients reported as having the juvenile phenotype and a single patient as infantile/late-infantile. The third most frequent genotype was p.P428L/p.P428L (*n* = 24; 4.9%) with 15 patients having juvenile phenotypes and 9 patients having adult phenotypes. For the 6 patients with this genotype and age of onset information, we observed that the average age of onset was 15.3 years, close to the cut-off in age ranges between the adult and juvenile forms of the disease (16 years).

### Individual variants observed in the patient data set

The frequency of each mutation found in the curated patient data set is provided in Additional file 3: Table S3, which also includes variants from gnomAD, variants from patients in the MLD Foundation registry, and NBS. In 40 cases from the curated patient data set, the second allele was not reported, resulting in 932 curated patient alleles for analysis. The curated patient data set contained 246 unique variants, although 2 variants accounted for 30% of all observed alleles: the splicing mutation c.465 + 1G > A (*n* = 186; 20.0%) and the missense mutation p.P428L (*n* = 117; 12.6%). p.I181S was the third most common allele in patients (*n* = 25; 2.7%).

### Allele frequency of pathogenic variants from the patient data set in gnomAD

To further analyze the curated patient data set, we extracted the allele frequency of each mutation found in gnomAD. Frequencies by population are shown in Additional file 3: Table S3. We observed that while only 26.8% of the 246 unique variants in patients were in gnomAD, these variants represent the majority of alleles (65.2%) reported in curated patients (612 of 938). Among variants in curated patients, p.T393S had the highest allele frequency in gnomAD, with an overall allele frequency of 0.48. p.T393S was also the major allele in both the Finnish (0.59) and the Non-Finnish European (NFE) populations (0.54). Although this mutation was observed in 11 MLD patients, p.T393S is a well-documented pseudo-deficiency variant in *ARSA*, with a high allele frequency, and is annotated as benign in ClinVar [11]. The three most common pathogenic mutations in the curated patient data set—c.465 + 1G > A, p.P428L, and p.I181S—were the three most common pathogenic mutations in gnomAD. The most common, c.465 + 1G > A, had an overall allele frequency of 6.4 × 10^−4^ and an NFE frequency of 1.2 × 10^−3^. p.P428L and p.I181S were the second and third most frequent variants in gnomAD, with NFE frequencies of 6.3 × 10^−4^ and 3.7 × 10^−4^, respectively. These data highlight the fact that, although many variants found in patients are not in gnomAD, such variants are rare, both in the overall population, and in patients. Conversely, while representing a small fraction of the unique variants found in patients, most patients will have mutations found in gnomAD. Furthermore, the most frequent alleles in the patient dataset are also the most common pathogenic alleles in the general population, as reported in gnomAD.

It may be noted that gnomAD is depleted of patients with pediatric diseases, but population allele frequencies of variants involved in autosomal recessive disease are determined mostly by the frequencies at which they occur in carriers. Allele frequencies should therefore be well determined by gnomAD.

### ClinVar annotation of identified *ARSA* variants

ClinVar pathogenicity annotations are shown in Additional file 3: Table S3. “Uncertain significance” was the most common ClinVar annotation among mutations from curated patients, gnomAD, and other sources (*n* = 39). A further 349 mutations were absent from ClinVar, with 24 variants annotated as “not provided” and 1 as “other,” leading to a total of 413 variants classified as VUS (clinical effects unknown).

ClinVar contained 58 *ARSA* variants that were labeled as pathogenic (31 pathogenic, 16 pathogenic / likely pathogenic, 11 likely pathogenic), 12 benign *ARSA* variants (2 benign, 8 likely benign, 2 benign / likely benign), and 14 variants annotated as having conflicting interpretations of pathogenicity. Of the latter, p.P220L (with a frequency of 0.028, and 10 homozygous individuals in the Finnish population in gnomAD) and c.466-7G > C (with a frequency of 0.004 in the NFE population in gnomAD) were considered to be benign in our analysis based on these high frequencies and their absence from the patient data set.

### Classifying variants using the curated patient data set: patient-based variant severity rules

**Fig. 2:**
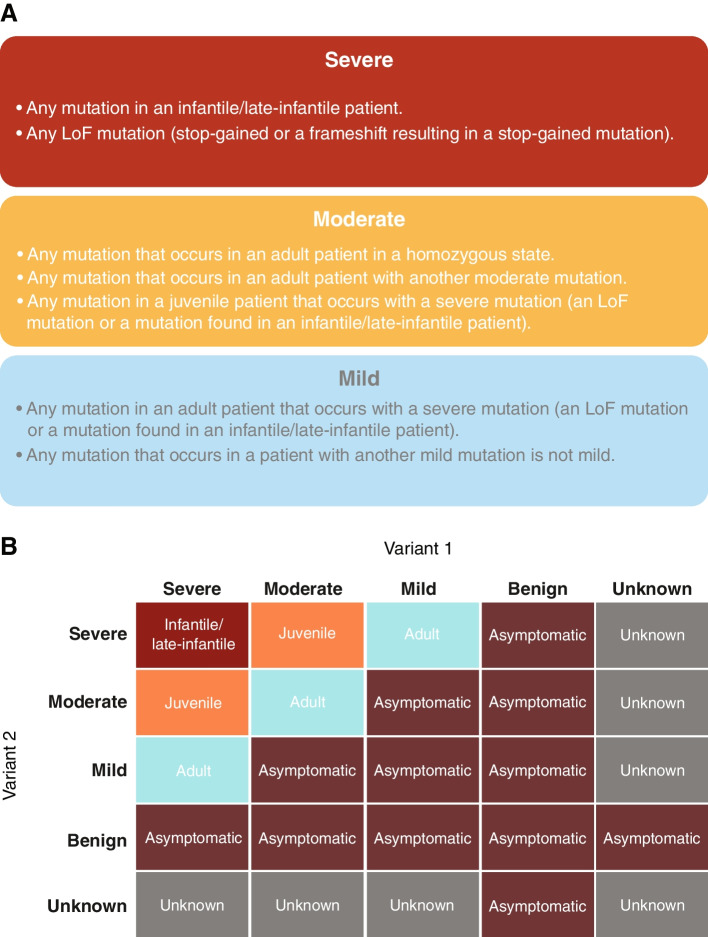
The patient-based severity ruleset and the phenotype matrix. **A** Ruleset for determining patient-based severity of *ARSA* variants. **B** The phenotype matrix used for prediction of phenotype from variant severity. The exterior labels of this matrix represent the severity assignments (severe, moderate, mild, benign, unknown) for each variant in the genotype. The interior cells represent the expected MLD phenotype (infantile/late-infantile, juvenile, adult, asymptomatic, unknown) produced by the combination of variants. ARSA, arylsulfatase A

The patient data set revealed that individuals with identical genotypes typically exhibit the same severity phenotype (i.e., similar age of onset of disease). Genotypes and phenotypes from the curated patient data set were used to derive a ruleset for classifying variant severity (Fig. 2A). In order to capture the varying levels of impact on ARSA enzyme activity, variants were grouped into four categories: severe, moderate, mild, and benign. Patient-based severity assignments were limited to mutations observed in 5 or more patients to ensure robust classification. Any mutation found in an infantile/late-infantile patient was described as severe. Loss of function (LoF) mutations (stop-gained or out-of-frame insertions/deletions leading to a stop-gained mutation) were classified as severe since these almost universally eradicate ARSA enzyme activity (an exception might be a terminal, stop-gained mutation) [13]. In this way, we constructed an initial list of severe mutations. During examination of reported juvenile MLD patients, any mutation found in *trans* with a severe mutation was classified as moderate under the assumption that a second severe mutation would have resulted in infantile/late-infantile MLD. Mutations in adult MLD patients with a homozygous genotype, or cases where an unclassified mutation was in *trans* with an already categorized moderate mutation, were classified as moderate as well. This was based on the hypothesis that a homozygous mild mutation would likely cause an asymptomatic phenotype, and homozygous severe mutations would cause the infantile/late-infantile form of the disease. This defined the list of moderate mutations. Among adult MLD patients, if a severe mutation was present, the second mutation was classified as mild, under the hypothesis that if it were moderate, it would result in juvenile MLD. Finally, although we did not curate asymptomatic individuals, MLD is an autosomal recessive disease, so any variant paired with a severe/moderate/mild mutation in a person who never develops MLD is considered benign. In our description of the phenotype matrix, we also point out that individuals with mild/mild or mild/moderate genotype combinations could be expected to be asymptomatic or have very late or subtle onset of symptoms.

**Fig. 3:**
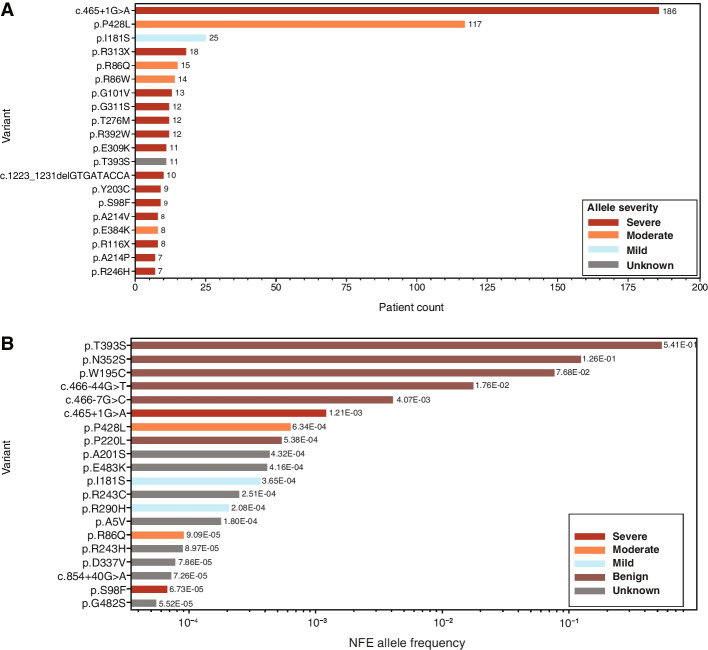
The most prevalent *ARSA* variants. **A** The 20 most prevalent *ARSA* variants in the curated patient data set, according to patient-based severity assignments. **B** The 20 most prevalent *ARSA* variants in the gnomAD NFE population, according to patient-based severity assignments. ARSA, arylsulfatase A; NFE, non-Finnish European

Again, patient-based severity assignments were limited to mutations observed in 5 or more patients to ensure robust classification (37 *ARSA* mutations met this requirement, representing 610 of 932 alleles [65.5%] reported in patients). These variants of known severity are depicted in Fig. 3. Of these, 28 (75.7%) were annotated as severe, representing 43.2% of alleles (*n* = 403) found in the curated patient data set. Moderate variants comprised 5 distinct alleles (13.5%), but these represented a larger percentage of alleles (*n* = 159; 17.1%) found in patient genotypes. Mild mutations comprised 5.4% alleles (*n* = 2), representing 3.3% of alleles (*n* = 31) found in patient genotypes. Finally, 5.4% of mutations (*n* = 2) observed in 5 or more MLD patients were documented as benign and observed in 17 patient genotypes (1.8%). In cases where fewer than 5 patients were reported, we assigned a patient-based severity of “unknown” and excluded these from genotype–phenotype analysis. The most prevalent variants in the curated patient data set and the gnomAD NFE population are shown in Fig. 3A and B, classified according to patient-based severity assignments.

When categorizing variant severities, we started with the most common variants and genotypes observed in patients (Table 1 and Figs. 1 and 3). The most common allele in patients is the c.465 + 1G > A splicing variant, found in curated patients 186 times. Patients homozygous for this allele typically presented with infantile/late-infantile MLD (*n* = 32), although one patient had an unknown phenotype. p.P482L was the second most frequent mutation in patients (*n* = 117). Within the data set, p.P428L homozygotes exhibited adult (*n* = 9) and juvenile (*n* = 15) forms of MLD, with an average age of onset (15.3 years) near the 16-year cutoff between the adult and juvenile forms of the disease. When p.P428L was paired with the severe mutation c.465 + 1G > A, patients mainly presented with juvenile MLD. There was one patient reported as having infantile/late-infantile MLD with genotype p.P428L/c.465 + 1G > A [4]. These data supported the assignments of severe for c.465 + 1G > A and moderate for p.P428L. Finally, the third most frequent *ARSA* mutation was p.I181S, appearing 25 times in curated patients. No p.I181S homozygotes were found in the MLD literature, nor was it observed in the compound heterozygous state with moderate p.P428L.Combined with c.465 + 1G > A, p.I181S produced the adult phenotype 4 times, the juvenile phenotype 4 times, and the infantile/late-infantile phenotype once. This supported the creation of a variant severity that is less severe than moderate, i.e., the mild severity. The most common genotypes associated with each predicted phenotype are shown in Fig. 1B and Table 2.

**Table 1:** Most common variants in the curated patient dataset

Reference SNP ID	Genomic coordinate GRCh37	HGVSc	HGVSp	Patient count, n	ClinVar pathogenicity	Patient-based severity	CDS activity-based severity	CDS percentage of wild-type activity, %	Overall gnomAD frequency	gnomAD population maximum frequency	gnomAD population
rs80338815	chr22:51065593:C > T	c.465 + 1G > A	NA	186	Pathogenic	Severe	NA	NA	6.36E − 04	1.21E − 03	NFE
rs28940893	chr22:51063820:G > A	c.1283C > T	p.P428L	117	Pathogenic	Moderate	Severe	0.037	4.08E − 04	9.47E − 04	Other
rs74315457	chr22:51065404:A > C	c.542 T > G	p.I181S	25	Pathogenic	Mild	Mild	4.32	2.26E − 04	4.06E − 04	Finnish
rs551472773	chr22:51064624:G > A	c.937C > T	p.R313X	18	Pathogenic	Severe	NA	NA	1.26E − 05	2.79E − 05	NFE
rs74315458	chr22:51065802:C > T	c.257G > A	p.R86Q	15	Pathogenic	Moderate	Moderate	2.13	8.09E − 05	2.74E − 04	Finnish
rs199476352	chr22:51065803:G > A	c.256C > T	p.R86W	14	Conflicting interpretations of pathogenicity	Moderate	Mild	7.86	NA	NA	NA
rs74315455	chr22:51065757:C > A	c.302G > T	p.G101V	13	Pathogenic	Severe	Severe	0.069	NA	NA	NA
rs74315459	chr22:51064630:C > T	c.931G > A	p.G311S	12	Conflicting interpretations of pathogenicity	Severe	Severe	0.28	2.92E − 05	9.94E − 05	South Asian
rs74315480	chr22:51064043:G > A	c.1174C > T	p.R392W	12	Conflicting interpretations of pathogenicity	Severe	Severe	0.20	1.63E − 05	6.50E − 05	South Asian
rs74315472	chr22:51065046:G > A	c.827C > T	p.T276M	12	Pathogenic / likely pathogenic	Severe	Severe	0.098	4.07E − 06	1.82E − 04	Other
rs743616	chr22:51064039:G > C	c.1178C > G	p.T393S	11	Benign	Benign	Benign	14.69	4.82E − 01	5.93E − 01	Finnish
rs199476360	chr22:51064636:C > T	c.925G > A	p.E309K	11	Pathogenic	Severe	Severe	1.36	4.14E − 06	5.87E − 05	East Asian
rs74315481	chr22:51063872:TGGTATCAC > -	c.1223_1231delGTGATACCA	p.S408_T410del	10	Pathogenic / likely pathogenic	Severe	Severe	0.23	8.53E − 06	9.50E − 06	NFE

*CDS* Coding sequence, *gnomAD* Genome Aggregation Database, *HGVSc* Human Genome Variation Society coding variant, *HGVSp* Human Genome Variation Society protein variant, *NA* Not applicable, *NFE* Non-Finnish European, *SNP* Single-nucleotide polymorphism

**Table 2:** Most common genotypes in the curated patient dataset and observed phenotypes. Entropy is a measure of phenotypic variability for a genotype

Genotype	Patient count, *n*	Percentage of patients, %	Phenotype breakdown	Entropy
c.465 + 1G > A/c.465 + 1G > A	33	6.75%	• Infantile/late-infantile 32 • Unknown 1	0.14
c.465 + 1G > A/p.P428L	26	5.32%	• Juvenile 25 • Infantile/late-infantile 1	0.16
p.P428L/p.P428L	24	4.91%	• Juvenile 15 • Adult 9	0.66
c.465 + 1G > A/p.I181S	9	1.84%	• Adult 4 • Juvenile 4 • Infantile/late-infantile 1	0.96
p.R313X/p.R313X	7	1.43%	• Infantile/late-infantile 6 • Unknown 1	0.41
c.465 + 1G > A/p.R290H	5	1.02%	• Adult 3 • Juvenile 2	0.67
c.465 + 1G > A/p.R86W	5	1.02%	• Juvenile 5	0.00
c.465 + 1G > A/c.855-1G > A	4	0.82%	• Infantile/late-infantile 4	0.00
c.465 + 1G > A/p.R86Q	4	0.82%	• Infantile/late-infantile 2 • Juvenile 2	0.69
p.R86Q/p.S98F	4	0.82%	• Adult 2 • Juvenile 2	0.69

### Development of a phenotype matrix

The phenotype matrix formalizes the genotype–phenotype relationship as a look-up table based on the assumption that the impact of each *ARSA* allele on enzymatic activity is additive (Fig. 2B). The impact of each *ARSA* allele on disease severity is also additive, with combinations of the most severe mutations causing the earliest onset form of disease, and each increase in ARSA enzymatic activity producing a milder, later onset phenotype.

The matrix classifies patients as infantile/late-infantile, juvenile, adult, or asymptomatic based on the classification of their alleles as severe, moderate, or mild. We noted that adult MLD generally resulted from pairing mild variants, such as p.I181S, with a severe allele, such as c.465 + 1G > A, and so we proposed that pairing any mild mutation with a less severe variant (moderate or mild) should result in a less severe phenotype than adult (probably not symptomatic for MLD). Therefore, the asymptomatic phenotype was also included, resulting from the pairing of mild mutations with moderate, mild, or benign mutations. In the curated patient data set, two mutations, p.I181S and p.R290H, consistently resulted in adult or juvenile MLD when paired with severe mutations, such as c.465 + 1G > A. However, p.I181S and p.R290H were never found with p.P428L or p.R86Q, which are moderate mutations. Furthermore, p.I181S and p.R290H never occurred with themselves or each other in symptomatic patients in the patient data set.

When a VUS is encountered, the phenotype can only be predicted by our matrix if the second allele is benign or wild-type. As such, four combinations of VUS-containing genotypes produce unpredictable phenotypes in the matrix. This highlights the importance of characterizing VUS to decrease the uncertainty in genotypes. Following our patient-based severity analyses, many VUS remained; the strategy to further characterize VUS in this study was to measure the enzyme activity of ARSA protein encoded by VUS-containing *ARSA* transcripts in a cell model.

### Selection of *ARSA* variants for activity assays

Activities of mutations (*n* = 37) that occurred in patients five or more times and had been assigned patient-based severity annotations (“severe” *n* = 28, “moderate” *n* = 5, “mild” *n* = 2, “benign” *n* = 2) were used to determine the range of ARSA enzyme activity that defines each patient-based severity. Then, the predicted variant severity of any given VUS (expressed alone in the *ARSA* consensus coding sequence in HEK293T cells) was determined by the bracket of ARSA enzyme activity in which the result fell. We assayed the activity of all 230 missense mutations in *ARSA* listed in gnomAD.

Of these mutations, 24 were annotated as pathogenic in ClinVar. One in-frame deletion (c.1223_1231delGTGATACCA) and one stop-gained mutation (p.C158X) were also selected to demonstrate the ability of the assay to characterize other classes of variants. We also included 29 variants from the literature that were not present in gnomAD, and 20 novel mutations from unpublished patients provided by MLD Foundation (including two stop-gained mutations: p.C71X and p.Y41X), resulting in a total of 281 coding sequence variants. Of the variants selected for testing, 82 mutations occurred in MLD patients at least once.

Variants for testing within the genomic construct (ENST00000216124.5) were prioritized by selecting suspected splice mutations from the literature (*n* = 21), along with hits from the splice prediction algorithm MaxENT. We were able to select a limited subset of variants (*n* = 8) that MaxENT classified as gain-of-splice acceptors, with > 40% difference between wild-type and mutant scores. Any missense variant within 6 bases of a splice junction (*n* = 3) was also tested in the genomic *ARSA* construct.

### ARSA enzyme activity assay

Assay results and enzymatic activity for each variant are listed in Additional file 3: Table S3. Briefly, CRISPR/Cas9 [clustered regularly interspaced short palindromic repeats / CRISPR‑associated protein 9] editing was used to create a clonal *ARSA*‑knockout line in HEK293T cells (knockout across all alleles was verified; Additional file 4: Fig S1). This cell line was used to test the 281 variants within the *ARSA* coding construct. Of the variants tested in the assay, 26 were classified as pathogenic, 5 were classified as benign, and 251 were categorized as VUS according to the patient-based severity assignments described previously.

**Fig. 4:**
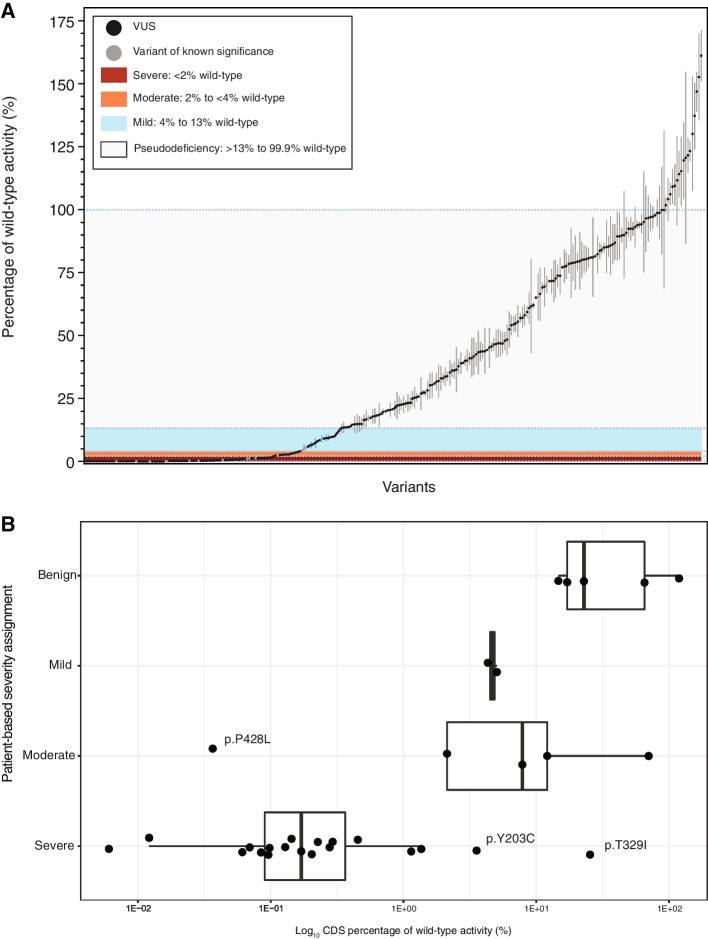
ARSA enzyme activity. **A** CDS enzymatic activities of ARSA variants. Percent of wild-type CDS enzymatic activities normalized to BLA levels for all 281 variants are plotted along with standard deviations (based on measurement of three separate wells of transfected HEK293T cells per variant). Brackets of activity-based severity are delimited by blue dashed lines. **B** ARSA enzymatic activity by patient-based severity categories. Box and whisker plot of CDS ARSA activity displayed by variants classified as benign, mild, moderate, or severe by the patient-based severity ruleset. Boxes indicate the interquartile range, with the median shown as a vertical line, and whiskers indicate the full range of data. ARSA, arylsulfatase A; CDS, coding sequence

We measured the enzyme activity of ARSA and normalized to beta-lactamase (BLA) activity as per the “Methods.” This is expected to minimize inaccuracies due to variation of transfection efficiencies among the different wells of transfected cells. In each set of enzyme assays, we also included wells of cells transfected with wild-type *ARSA* (to minimize assay drift over time), and the percentage of wild-type activity for each ARSA variant was calculated as 100 × [(ARSA variant activity)/(BLA activity in ARSA variant cells)]/[(ARSA wild-type activity)/(BLA activity in ARSA wild-type cells)]. Percent of wild-type activities are displayed in Additional file 5: Table S4 for coding sequence (CDS) variants, and Fig. 4A shows enzyme activities of all 281 *ARSA* CDS variants measured in HEK293T cells ordered by increasing activity. Normalization to BLA activity presumably accounts for any differential transfection efficiency among the different variants tested. For precision, enzymatic activity is reported as directly calculated and may include negative values, due to the nature of blank subtractions. Variant activity can be negative when the enzymatic activity is close to zero and gives an observed value slightly lower than observed with the blank in which only cell lysis buffer was used. For the purpose of genotype–phenotype analysis, negative values reflect null enzymatic activity.

### Classifying variants using activity data: activity-based severity

Using the CDS percentage of wild-type activity of pathogenic mutations from the curated patient data set, we set 13% of wild-type activity as the upper limit for defining pathogenic variants in our assay, based on variants p.I181S and p.T393S. p.I181S, annotated as mild in 25 patients, exhibited the lowest activity in this class (4.3% of wild-type) and helped set the lower bound for mild mutations. p.T393S, reported as benign in ClinVar, had the lowest activity in the benign class (14.7% of wild-type), informing the upper bound for mild severity. Enzyme activity thresholds for each variant severity category were derived in the same manner (Fig. 4A); severe activity limits were set at 0 to < 2% of wild-type activity, moderate limits were set at 2 to < 4% of wild-type activity, and mild limits were set at 4 to 13% of wild-type activity.

Of the 251 variants with unknown patient-based severity assignments tested in the activity assay, we observed that 90 (36%) had activity values below 13%, and could potentially be considered as pathogenic. Furthermore, 65 (26%) of the interrogated VUS displayed ARSA activity in the 0–3% range, and are thus predicted to be severe.

### Enzyme activities of variants in the genomic construct

Also assayed were a subset of *ARSA* variants with an intron-inclusive expression plasmid, to demonstrate the ability of the model to quantify splice mutations. We assayed 32 mutations in a genomic *ARSA* construct. Variant activity as a percentage of wild-type activity is displayed in Additional file 6: Table S5.

This set of mutations in the genomic construct included 29 VUS and 3 well-characterized variants (2 severe and 1 moderate by patient-based severity), observed in 5 or more patients in the literature.

To examine continuity between assays, four variants (p.A325T, p.H228D, p.E384K, and p.S227C) were tested in both CDS and genomic constructs. Activity-based severity agreed between CDS and genomic constructs for three of these variants. However, for p.S227C, genomic activity-based severity differed from the activity-based severity assigned by CDS activity (benign by genomic activity-based severity compared with severe by CDS activity-based severity). p.E384K was a pathogenic (moderate) variant according to patient-based severity and ClinVar, but displayed CDS and genomic activities in the benign range. p.E384K, implicated as a splice variant by MaxENT, exhibited 70.2% of wild-type activity in the CDS assay but 18.3% of wild-type activity in the genomic assay. Although both activity levels fall in the benign category, the almost fourfold lower activity of the genomic construct suggests that splicing may play a role in its pathobiology.

Finalized severity was determined by comparing CDS activity to genomic (when available) activity-based severity and the patient-based severity, conceding to the general consensus in the literature if there were any disagreements between activity values. If CDS activity-based severity did not equal the genomic activity-based severity, the finalized severity was set to the activity-based severity that most closely matched the patient-based severity/literature. In the case of p.P428L, we set the finalized severity to the patient-based severity, since this mutation had unique properties.

### Agreement of enzyme activity and patient-based severity

In our HEK293T studies, 116 variants were identified with activity values in the range of what could be considered pathogenic (CDS activities ≤ 13% of wild-type activity) along with 165 variants whose activities were above this threshold and could be considered benign (Additional file 5: Table S4). The CDS activity brackets described previously were applied to 251 VUS, yielding 161 benign, 66 severe, 9 moderate, and 15 mild predictions. As described above, 37 variants from the literature documented in 5 or more individuals were assigned a patient-based severity, 31 of which were tested in the CDS enzyme activity assay.

Patient-based severity and CDS activity-based severity classifications agreed for 25 of these 31 mutations (Fig. 4B). The ARSA enzyme activity assay could distinguish not only between pathogenic and benign mutations, but also between mild, moderate, and severe mutations with reasonable, but not perfect, accuracy. Overlap between categories may be an artifact of normalization or due to well-to-well variation. For each variant in the full data set (Additional file 3: Table S3), a cross-tabulation of annotations from ClinVar is provided with subsequent patient-based severity and ARSA activity-based severity assignments (Additional file 8: Table S7). Of variants characterized in five or more patients, the CDS assay misclassified six variants: p.P379L, p.R86W, p.P428L, p.E384K, p.Y203C, and p.T329I. p.E384K and p.T329I were the only pathogenic variants to possess high CDS activity (70.2 and 25.4% wild-type activity, respectively). p.E384K also tested as benign using the genomic construct, with 18.3% of wild-type activity. This mutation was observed 8 times in 7 different patients with 6 different genotypes. One p.E384K homozygote was reported and exhibited juvenile-onset MLD. Analysis by MaxENT suggests that this mutation affects splicing, which may explain the decreased activity in the genomic assay, but p.E384K did not exhibit moderate activity, as its patient-based severity annotation suggests. Possibly, the effect of p.E384K on splicing efficiency has a cell type-specific component that is incompletely observed in the HEK293-based assay. A similar finding was observed for p.R372Q, which has been annotated as pathogenic/likely pathogenic in ClinVar, but as having an unknown patient-based severity. It occurred six times in curated patients, but in one instance was paired with an unidentified second mutation. Based on its occurrence in late-infantile patients, it would be considered a severe mutation. It displayed 23.32% of wild-type activity in the CDS assay. The variant occurs about 8 nucleotides downstream of an acceptor site and may affect splicing; therefore, p.R372Q should be better assessed with the genomic construct.

The remaining four misclassified pathogenic mutations from the curated patient data set had less than 13% of wild-type levels. p.R86W was classified as mild (7.9% of wild-type activity) but is considered moderate under our patient-based severity ruleset. In ClinVar, this mutation was listed as “conflicting interpretations of pathogenicity,” consistent with the variable onsets of 11 individuals in the patient data set. p.P379L (12.1% of wild-type activity) was the only other moderate mutation in the literature that was classified as mild in the enzyme activity assay.

Furthermore, c.684 + 1G > A, a known pathogenic variant, showed 48.6% of wild-type activity using the genomic construct. While this variant did not occur in our curated patient data often enough to be assigned a patient-based severity, it is annotated as pathogenic in ClinVar. This again suggests that there are additional factors affecting enzyme function or splicing efficiency in disease-relevant cells that are not recapitulated in our HEK293T cell model. In addition, the genomic construct assay was less robust than it CDS counterpart, which was likely due to cell toxicity/death and/or reduced transfection/expression levels.

p.P428L, the second most frequent variant found in patients, was also considered moderate in the reported patient-based severity literature. Its final activity-based severity was set to severe, with 0.04% of wild-type CDS activity. The results of the p.P428L CDS activity were similar to previous reports using constructs bearing *ARSA* complementary DNA (cDNA) [9, 11]. This mutation exhibited an interesting biochemical pattern. The lysosomal protease cathepsin L does not cleave wild-type ARSA but does cleave the p.P428L variant near the site of mutation [15]. Thus, the activity of the variant would be predicted to be dependent on expression of cathepsin L. There could be cell type-specific proteases that mediate enzyme activity in various tissues and cell types. The mismatch between the moderate patient-based severity of p.P428L and the extremely low enzyme activity of this variant in HEK293T cells remains unexplained.

On a side note, 59 *ARSA* variants were classified as pathogenic, likely pathogenic, benign, or likely benign in ClinVar, among which 52, or 88%, had activity-based severities that agreed with the curation if a cutoff of 13% of wild-type activity was used to distinguish between pathogenic and benign variants.

We calculated the diagnostic odds ratio (DOR) of our enzymatic assay using the CDS activity and patient-based severity classifications for variants that occurred 5 or more times in patients or were known to be benign [16]. Because our screening strategy, with a threshold of 13% activity to discriminate between pathogenic and benign variants, resulted in no false positives, our likelihood ratio (LR +) and DOR were infinite. This implies that the enzymatic activity assay reliably detects pathogenic variants, and possesses a specificity or true‑negative rate of 100%. It is important to note that the 13% threshold did result in two false negative variants that are classified as pathogenic but have high activity. Combining genetic and biochemical data increases diagnostic yield and the ability to accurately diagnose patients. For instance, false negatives from the biochemical assay, or known pathogenic variants that exhibit high activity, can be reclassified using patient-based severity rules and the phenotypes of patients genotyped by NGS. Additionally, VUS for which no pre-existing patient data exist can be characterized by a biochemical assay.

### Correlation of *in silico* predictors with VUS enzyme activities

The measured ARSA variant enzyme activities in HEK293T cells were compared to those predicted by three in silico methods: SIFT [17], PolyPhen [18], and REVEL [19]. *ARSA* variants characterized in ClinVar were excluded from this analysis since ClinVar is used to train SIFT and PolyPhen predictions (Additional file 3: Table S3 and Additional file 4: Fig S2). Correlations between CDS enzyme activity data with SIFT, PolyPhen, and REVEL (*n* = 219) were low, with Pearson’s R correlation coefficients of 0.44, − 0.56, and − 0.51, respectively (Table 3). These results were in accordance with other examinations of in silico methods [20–22] and demonstrate the need to understand the impact of variants using other techniques, such as our high-throughput, HEK293T cellular, and enzyme activity assay. In general, these in silico methods are not suitably accurate for use in prognosis of disease, especially in asymptomatic newborns.

**Table 3:** Pearson’s R correlation coefficients between CDS wild type activity and SIFT, PolyPhen, and REVEL predictor scores. CDS, coding sequence

	**CDS mean percent of wild-type activity**	**SIFT scores**	**PolyPhen scores**	**REVEL scores**
CDS mean percent of wild-type activity	1	0.44	− 0.56	− 0.51
SIFT scores	0.44	1	− 0.67	− 0.64
PolyPhen scores	− 0.56	− 0.67	1	0.86
REVEL scores	− 0.51	− 0.64	0.86	1

### Assessment of the genotype–phenotype relationship using entropy calculations

The strength of the genotype–phenotype relationships generated in this study was evaluated in multiple ways. First, we measured the entropy, or the amount of uncertainty associated with common genotypes (Additional file 2: Table S2). For this analysis, we used genotypes that occurred five or more times in curated patients. There were 9 such genotypes, representing 28.3% of curated patients (*n* = 138). We used entropy as a measure of the consistency of the genotype–phenotype relationship, which was calculated as follows:

$$H\left[G\right]=\sum_{i=1}^{n}\widehat{\mathrm{p}}\left({Phenotype}_{i}|Genotype\right)\times \mathrm{log}\left(\widehat{\mathrm{p}}\left({Phenotype}_{i}|Genotype\right)\right)$$

Here, $\widehat{\mathrm{p}}$(*Phenotype*_*i*_|*Genotype*) was the probability of phenotype* i* given the specific genotype being analyzed, and* i* ranges from 1 to *n* distinct phenotypes associated with the genotype. A genotype that consistently presented with the same phenotype had an entropy of zero, whereas genotypes that presented with multiple phenotypes had higher entropies. For this analysis, phenotypes were classified as infantile/late-infantile, juvenile, and adult, consistent with phenotype designations used throughout this analysis. This categorization resulted in a maximum permitted entropy of 1.099. For genotypes with both alleles documented in 5 or more patients, the mean entropy was 0.099, with a standard deviation of 0.241. While this average entropy is not trivial, it is near the ideal entropy of zero, suggesting high genotype–phenotype consistency in MLD.

The c.465 + 1G > A/p.I181S genotype (*n* = 9) had the highest entropy (0.96). At least one case in the literature with this genotype was reported to have late-infantile MLD but was noted by the authors to have a “pre-existing neurological comorbidity” [23]. At the other extreme were the c.465 + 1G > A/c.465 + 1G > A (*n* = 33) and c.465 + 1G > A/p.P428L (*n* = 26) genotypes, which were the most consistent and most frequent genotypes, with entropies of 0.14 and 0.16, respectively. The p.P428L/p.P428L genotype (*n* = 24) was less consistent (entropy: 0.66), with 9 curated patients annotated as having adult MLD and 15 with juvenile disease. Although there was some inconsistency with this genotype, for patients with age of onset data (*n* = 6) mean onset was 15.3 years. This is higher than the average age of onset for the c.465 + 1G > A/p.P428L genotype (*n* = 14; mean: 5.30 years) and consistent with the prediction of the phenotype matrix that the latter genotype is more severe (i.e., has an earlier onset) compared with the p.P428L/p.P428L genotype.

### Accuracy of genotype–phenotype analysis using the phenotype matrix

**Fig. 5:**
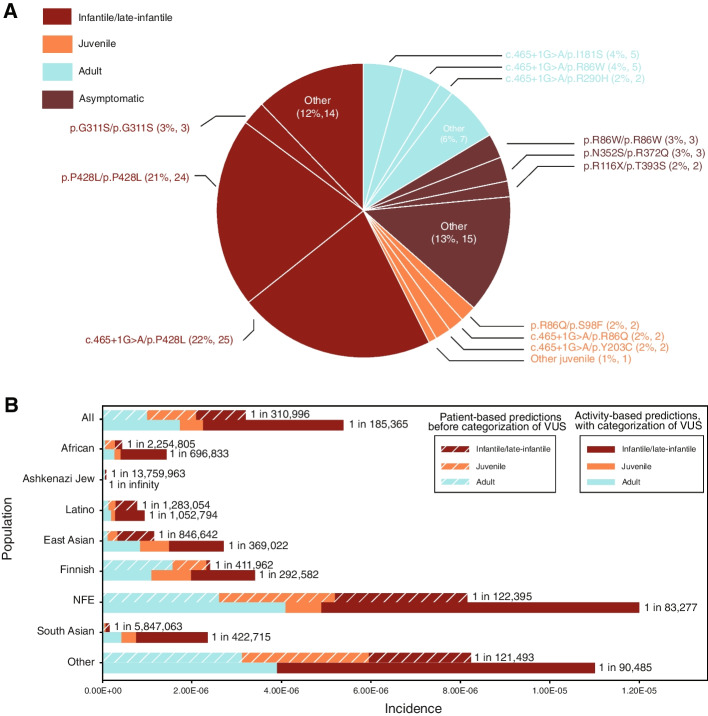
Frequently inconsistent MLD genotypes and estimated burden of MLD. **A** Summary of the genotypes whose phenotypes did not match the patient data set and were predicted incorrectly using the phenotype matrix. These genotypes manifested with multiple phenotypes within the patient data set. We considered genotypes where each mutation occurred at least five times in the overall patient data set and ignored any patient where two mutations were not identified. We then considered the predicted phenotype based on the finalized severity of each mutation. For each genotype, we counted the number of times the predicted phenotype agreed with the observed phenotype. **B** Classifying VUS improves the accuracy of MLD disease burden estimates using allele frequencies from multiple subpopulations in gnomAD. This is demonstrated by estimation of the burden of MLD in a bar chart including the proportion of each MLD subtype. These calculations were carried out before (light bars) and after (saturated bars) VUS were characterized for ARSA enzymatic activity in HEK293T cells. Classifying VUS decreased the number of GUS, improving the estimates of disease burden. ARSA, arylsulfatase A; gnomAD, Genome Aggregation Database; NFE, non-Finnish European; VUS, variants of unknown significance

The accuracy of the phenotype matrix in predicting phenotypes from the severity of each mutation in a patient’s genotype was also analyzed (Additional file 2: Table S2 and Fig. 5A). Analysis was limited to genotypes for which each mutation occurred five or more times in patients, to ensure that the underlying patient data were reproducible and accurate. Although 46.7% of MLD patients (*n* = 227) had unique genotypes, a similar percentage of curated patients had genotypes where both mutations were found in the patient data five or more times (*n* = 233, represented by 77 distinct genotypes) (Additional file 2: Table S2). These genotypes were utilized along with the phenotype matrix to evaluate the strength of the genotype–phenotype relationships in MLD. For each genotype, the phenotype matrix was used to generate a predicted phenotype given the severity of each mutation. The accuracy of these predictions was calculated as the percentage of observed phenotypes that matched the predicted phenotype for both patient-based severity and activity-based severity assignments. The accuracy of the phenotype matrix decreased with high-entropy genotypes from the patient data set, where the reported phenotypes varied widely. For example, c.465 + 1G > A/p.I181S had an entropy of 0.96 and a predicted phenotype of adult MLD. This genotype had 1 infantile, 4 adult, and 4 juvenile patients, giving it 44.4% accuracy for both patient-based severity and activity-based severity.

Using patient-based severity, the overall accuracy of the phenotype matrix was 76%, and using activity-based severity it was 52%. The discrepancy between the matrix’s patient-based severity and activity-based severity performance was largely attributed to misclassification of the frequent p.P428L mutation, observed in 117 patients (24.1% of individuals) across 29 distinct genotypes. As discussed previously, p.P428L was moderate under the patient-based severity rules but classified as severe in the CDS activity assay. If p.P428L-containing genotypes were excluded from the analysis, the overall patient-based severity accuracy of the phenotype matrix was 78%, but for activity-based severity it went from 52 to 70%. While not perfect, the phenotype matrix provides an additional tool that can be used to predict phenotype from genotype.

We acknowledge that calculating the accuracy of the phenotype matrix on the same set of patients used to determine variant severity may overestimate accuracy. However, it would be unfeasible to perform manual cross-validation to assess the accuracy of the rules for variant severity. In order to manually obtain an unbiased quantification of the strength of the genotype–phenotype relationship, variant classification for each random training set would need to be performed by separate individuals. To obtain an unbiased estimate of the accuracy of the phenotype matrix in MLD, we cross-validated a random forest model. In this model, we evaluated the performance of genotype information, CDS activity, and the two sources of information combined in predicting patient genotypes. Patient genotypes were one-hot encoded into a table with |V| columns, where |V| is the cardinality of the set of variants found in patients. The one-hot encoding of each individual variant in a patient’s genotype was summed, so that homozygous individuals had a 2 in the column corresponding to their respective variant, and heterozygotes a 1 in each respective column. Two features, each representing the CDS activity of each variant in a patient’s genotype, were also generated. In this representation, the CDS activity of stop-gained, frameshift, and the c.465 + 1G > A variant were set to 0.

We performed analysis after filtering patients in two ways. First, we filtered for only patients where each allele in the *ARSA* genotype occurred at least 5 times in the patient dataset. Second, we included patients where each variant in the genotype had an associated activity value from our assay or from setting the activity of stop-gained, frameshift, and c.465 + 1G > A variants to 0. The ability to predict an unseen patient’s phenotype was modeled by iteratively selecting 10% of patients as a test set with the remaining 90% being used to train a one-versus-rest multi-class random forest model in Python with each combination of the two feature sets. After 1000 iterations, the area under the curve (AUC) and accuracy were calculated across all testing folds. *P* values for calculating the statistical significance that one AUC is greater than another were calculated as the percentage of times the difference in AUCs were greater than that observed when iteratively shuffling prediction values between the two predictors.

When performing analysis on patients for whom each variant occurred at least 5 times in the dataset, we observed an AUC of 0.919 and an accuracy (the percentage of patients for which the correct phenotype had the highest score) of 75.4% when using genotype information alone. This value was very similar to the 76% observed when using patient-based severity annotations and the phenotype matrix manually. When using activity information only, we observed an AUC of 0.909, and an accuracy of 70.2%, much higher than the 52% obtained when using thresholds for mild/moderate/severe variant cutoffs and the phenotype matrix. As a sign of overfitting, we observed that when using activity information, the random forest model predicted the p.P428L/p.P428L genotype to have the infantile/late-infantile/, juvenile, and adult-onset phenotypes 0, 17, and 83% of the time respectively, although that genotype would be expected to have almost no residual activity according to the CDS assay. While clear evidence of overfitting, this is an example where both genotype and phenotype information can combine to increase the ability to predict a patient’s phenotype. Interestingly, combining both sets of features resulted in higher AUC (0.920), but slightly lower, accuracy than genotype information alone (73.7%).

When performing cross-validation with all patients for whom each variant has CDS activity, we found that the accuracy when combining each set of features (accuracy = 74.9%, AUC = 0.915) was higher than when only using genotype information (accuracy = 69.7%; AUC = 0.886, *P* < 0.001) or CDS activity (accuracy = 73.6%; AUC = 0.909, *P* < 0.001). We also observed that activity information had a higher AUC and accuracy than genotype information (*P* < 0.001). This is intuitive since this dataset is not filtered for patients for whom each variant occurs at least 5 times, and more patients in the test set will have variants not observed in the training set.

### Most common expected genotypes according to variant frequencies in gnomAD

The expected incidence of each combination of *ARSA* alleles was also calculated along with the predicted MLD phenotypes based on patient-based severity and activity-based severity (Additional file 9: Table S8). The expected incidence of an individual genotype was calculated as the product of the allele frequency of the two mutations comprising the genotype for each subpopulation reported in gnomAD. The most common genotypes associated with each predicted phenotype are shown in Fig. 1B﻿ and C.

c.465 + 1G > A/c.465 + 1G > A, the most common genotype in curated patients (*n* = 33), was also predicted to be the most common symptomatic genotype in the NFE population (NFE incidence = 1 in 683,188). The next two genotypes predicted to be pathogenic, c.465 + 1G > A/p.P428L and p.P428L/p.P428L (expected to occur with an NFE incidence of 1 in 651,761 and 1 in 2,487,121, respectively), were the next two most common genotypes in the curated patient data as well (*n* = 26 and 24, respectively). In accordance with the phenotype matrix, there were several genotypes that consisted of two pathogenic alleles (two mild variants or a mild and a moderate variant) that are predicted to be asymptomatic. The most common in this group was p.I181S/p.P428L, which was expected to occur with an NFE frequency of 1 in 2,159,790. This genotype was not observed in the curated data set. The genotypes p.I181S/pI181S and p.R86Q/p.I181S were expected to occur in 1 in 7,502,152 and 1 in 15,064,381, respectively, in the NFE population, and to date no patients with these genotypes have been reported. The combined expected NFE incidence of these three genotypes was 1 in 900,009**.** Given the ascertainment bias for NFE individuals in gnomAD, these allele frequencies influenced the confidence intervals of our incidence estimates.

### Estimates of disease burden and reduction in genotypes of uncertain significance

Patient-based severity and activity-based severity were used in combination with allele frequencies in gnomAD to predict the most common genotypes for each phenotype, including genotypes that are predicted to lead to a benign or unknown phenotype, and to estimate the incidence of MLD (Table 4). Frequencies for each genotype predicted to present with a particular subtype of the disease (infantile/late-infantile, juvenile, adult, asymptomatic, unknown) were summed to generate incidence estimates of each respective subtype. Ninety-five percent confidence intervals for each incidence estimate were also calculated as described in the “Methods.” We found the analytic method for calculating 95% confidence intervals closely matched values obtained through numeric simulation (Additional file 10: Table S9 and Additional file 4: Fig S3), and report those values in Table 4. Numeric, or bootstrapping, simulations were carried out as described previously, substituting the phenotype matrix method for calculating disease incidence [22].

**Table 4:** Predicted birth incidence of disease by population

Population	Adult	Juvenile	Infantile/late-infantile	Overall	Unknown	Asymptomatic
Patient-based severity frequencies						
All	1 in 982,195 (1 in 1,217,194–1 in 809,415)	1 in 922,918 (1 in 1,161,876–1 in 750,959)	1 in 897,800 (1 in 1,171,025–1 in 710,374)	1 in 310,996 (1 in 378,659–1 in 260,026)	1 in 11,635 (1 in 10,688–1 in 12,660)	1 in 2.0 (1 in 2.0–1 in 2.0)
African	1 in 14,176,960 (1 in 831,913,558–1 in 3,652,135)	1 in 4,702,212 (1 in 34,035,787–1 in 1,743,843)	1 in 6,238,516 (1 in 103,014,176–1 in 1,938,642)	1 in 2,254,805 (1 in 14,451,246–1 in 863,371)	1 in 1,500 (1 in 1,207–1 in 1,885)	1 in 2.0 (1 in 2.0–1 in 2.0)
Ashkenazi Jew	1 in 16,037,127 (1 in 5,420,710,450–1 in 3,467,993)	1 in Inf (1 in NA–1 in NA)	1 in 96,905,713 (1 in 103,489,540,292,125,616–1 in 11,904,593)	1 in 13,759,963 (1 in 8,104,690,917–1 in 2,848,333)	1 in 98,975 (1 in 52,193–1 in 229,712)	1 in 2.0 (1 in 2.0–1 in 2.0)
Latino	1 in 7,251,699 (1 in 34,320,883–1 in 3,034,293)	1 in 6,030,699 (1 in 26,581,588–1 in 2,582,041)	1 in 2,102,282 (1 in 5,968,435–1 in 1,061,892)	1 in 1,283,054 (1 in 3,111,363–1 in 696,030)	1 in 27,551 (1 in 21,067–1 in 37,271)	1 in 2.0 (1 in 2.0–1 in 2.0)
East Asian	1 in 8,241,012 (1 in 247,204,015–1 in 2,322,923)	1 in 4,499,667 (1 in 50,973,627–1 in 1,504,673)	1 in 1,193,952 (1 in 4,829,714–1 in 526,209)	1 in 846,642 (1 in 2,912,020–1 in 395,744)	1 in 3,775 (1 in 3,023–1 in 4,831)	1 in 4.0 (1 in 4.0–1 in 4.0)
Finnish	1 in 632,277 (1 in 1,473,092–1 in 349,708)	1 in 1,348,377 (1 in 4,014,767–1 in 667,509)	1 in 9,597,657 (1 in 112,527,413–1 in 3,186,559)	1 in 411,962 (1 in 934,766–1 in 230,856)	1 in 146,251 (1 in 89,716–1 in 274,619)	1 in 2.0 (1 in 2.0–1 in 2.0)
Non-Finnish European	1 in 383,549 (1 in 496,456–1 in 305,296)	1 in 385,798 (1 in 511,958–1 in 301,227)	1 in 336,590 (1 in 461,907–1 in 256,240)	1 in 122,395 (1 in 154,982–1 in 99,129)	1 in 32,069 (1 in 27,364–1 in 37,914)	1 in 2.0 (1 in 2.0–1 in 2.0)
South Asian	1 in 23,128,011 (1 in 230,477,824–1 in 7,950,208)	1 in 24,809,733 (1 in 344,874,636–1 in 7,961,844)	1 in 11,430,966 (1 in 85,649,278–1 in 4,202,324)	1 in 5,847,063 (1 in 27,595,032–1 in 2,448,746)	1 in 2,856 (1 in 2,434–1 in 3,377)	1 in 3.0 (1 in 3.0–1 in 3.0)
Other	1 in 321,219 (1 in 1,299,900–1 in 141,551)	1 in 350,719 (1 in 1,793,905–1 in 143,259)	1 in 441,214 (1 in 4,014,613–1 in 154,808)	1 in 121,493 (1 in 461,869–1 in 54,729)	1 in 7,989 (1 in 5,009–1 in 13,802)	1 in 2.0 (1 in 2.0–1 in 2.0)
Activity-based severity frequencies (95% CI)						
All	1 in 573,791 (1 in 710,318–1 in 473,265)	1 in 1,917,472 (1 in 2,816,519-1 in 1,389,872)	1 in 319,444 (1 in 391,416–1 in 265,699)	1 in 185,365 (1 in 220,577–1 in 157,985)	1 in 798,906 (1 in 605,837–1 in 1,128,508)	1 in 2.0 (1 in 2.0–1 in 2.0)
African	1 in 3,681,593 (1 in 39,388,957–1 in 1,246,120)	1 in 7,527,795 (1 in 453,677,291–1 in 1,932,964)	1 in 970,308 (1 in 3,884,229–1 in 429,195)	1 in 696,833 (1 in 2,382,388–1 in 326,467)	1 in 2,147,028 (1 in 671,520–1 in Inf)	1 in 2.0 (1 in 2.0–1 in 2.0)
Ashkenazi Jew	1 in Inf (1 in NA-1 in NA)	1 in Inf (1 in NA-1 in NA)	1 in Inf (1 in NA-1 in NA)	1 in Inf (1 in NA-1 in NA)	1 in 10,664,166 (1 in 2,142,447–1 in Inf)	1 in 2.0 (1 in 2.0–1 in 2.0)
Latino	1 in 5,080,870 (1 in 21,100,323–1 in 2,219,182)	1 in 10,340,390 (1 in 101,625,721–1 in 3,565,435)	1 in 1,523,627 (1 in 3,909,659–1 in 804,944)	1 in 1,052,794 (1 in 2,425,110–1 in 585,563)	1 in 2,892,784 (1 in 1,302,936–1 in 16,137,288)	1 in 2.0 (1 in 2.0–1 in 2.0)
East Asian	1 in 1,168,036 (1 in 3,548,349–1 in 573,449)	1 in 1,538,467 (1 in 5,990,990–1 in 687,123)	1 in 830,752 (1 in 2,851,688–1 in 388,610)	1 in 369,022 (1 in 930,942–1 in 196,496)	1 in 206,362 (1 in 116,392–1 in 453,224)	1 in 4.0 (1 in 4.0–1 in 4.0)
Finnish	1 in 904,289 (1 in 2,118,248–1 in 498,834)	1 in 1,139,390 (1 in 3,354,396–1 in 566,721)	1 in 697,186 (1 in 1,829,319–1 in 364,606)	1 in 292,582 (1 in 609,206–1 in 171,392)	1 in 282,508 (1 in 154,302–1 in 675,835)	1 in 2.0 (1 in 2.0–1 in 2.0)
Non-Finnish European	1 in 242,771 (1 in 314,762–1 in 192,988)	1 in 1,274,476 (1 in 2,322,899–1 in 804,303)	1 in 140,758 (1 in 181,201–1 in 112,521)	1 in 83,277 (1 in 103,413–1 in 68,514)	1 in 1,459,174 (1 in 865,972–1 in 3,501,638)	1 in 2.0 (1 in 2.0–1 in 2.0)
South Asian	1 in 2,326,525 (1 in 7,553,334–1 in 1,112,061)	1 in 2,965,978 (1 in 12,177,600–1 in 1,300,518)	1 in 625,516 (1 in 1,343,427–1 in 360,494)	1 in 422,715 (1 in 830,376–1 in 255,587)	1 in 2,276,709 (1 in 993,988–1 in 12,672,316)	1 in 3.0 (1 in 3.0–1 in 3.0)
Other	1 in 254,514 (1 in 1,258,278–1 in 105,052)	1 in Inf (1 in NA-1 in NA)	1 in 140,400 (1 in 602,201–1 in 60,658)	1 in 90,485 (1 in 311,412–1 in 42,285)	1 in 576,125 (1 in 150,023–1 in Inf)	1 in 2.0 (1 in 2.0–1 in 2.0)

Burdens for each MLD subtype were predicted using allele frequencies from gnomAD and our phenotype matrix. Calculations were performed using patient-based severity, as defined by our variant-severity criteria, and repeated using activity-based severity determined by tandem mass spectrometry (MS/MS). 95% percent confidence intervals were calculated using the analytical method, except for the “Unknown” and “Asymptomatic” incidence rates, which rely on 95% confidence intervals calculated through numeric simulation

Using patient-based severity assignments, the estimated incidence of MLD was 1 in 310,996 births when using overall allele frequencies (Fig. 5B). For the subpopulations defined in gnomAD, the estimated MLD frequency was higher in the NFE population (1 in 122,395) and lowest in the Ashkenazi Jew population (1 in 13,759,963). Using activity-based severity, the estimated overall incidence of MLD was 1 in 185,365, representing a 67.8% increase in the incidence based on patient-based severity. The estimated incidence of MLD in the NFE population based on activity-based severity was 1 in 83,277, representing a 47.0% increase in the incidence estimate based on patient-based severity assignments. For all other subpopulations in gnomAD other than the Ashkenazi population, an increased incidence of MLD was predicted when taking into account the additional information of VUS provided by activity-based severity assignments compared with patient-based severity putting into context the contribution of VUS to disease burden. Using activity-based severity assignments, the predicted incidence of MLD in the Ashkenazi Jew population was zero.

The predicted incidence of GUS or unknown phenotypes due to a genotype containing one or two *ARSA* VUS is especially important when one considers NBS for MLD among large populations. It was found that, in general, populations in which MLD was estimated to have the lowest incidence using patient-based severity assignments also had the highest estimated incidence of GUS (Fig. 5B and Additional file 7: Table S6). The exception to this was the Latino population which had a lower incidence rate than the East Asian population, but also had a lower incidence of GUS. The African subpopulation had the highest estimated rate of GUS (1 in 1500), whereas the Finnish subpopulation had the lowest estimated rate of GUS (1 in 146,251). It was estimated that the overall incidence of GUS was 1 in 11,636 using patient-based severity assignments.

With the use of activity-based severity assignments, there was a decrease in predicted GUS incidence. In all subpopulations in gnomAD other than the Finnish subpopulation, there was a > 97% decrease in the incidence of GUS (Additional file 7: Table S6). For the overall allele frequencies in gnomAD, the estimated incidence of GUS dropped from 1 in 11,636 births to 1 in 798,907 births when using activity-based severity assignments. The greatest decrease in GUS (99.9%) was found in the African subpopulation in gnomAD, with an estimated rate of 1 in 2,147,029.

### Contribution of mutations that are not present in gnomAD

As more individual genomes from the population are sequenced, more mutations will be identified in *ARSA*, yet new variants will be rarer and rarer, contributing little to the expected incidence of GUS in MLD. It may be argued that the combined frequency of singletons and unobserved variants in a population, given a set of mutations already identified in a reasonably large sample of sequenced individuals, is a function of the de novo mutation rate and the length of that gene. To put the contribution of unobserved mutations and singletons into context, the de novo mutation rate estimate for *ARSA* for missense (1.57E − 05) and splice site (4.04E − 07) mutations may be used [24]. Combined, these two mutation classes would be estimated to contribute de novo mutations to the population with a frequency of 1.61E − 05. In contrast, the combined allele frequency of missense VUS in gnomAD is 3.30E − 03.

While we have shown the incidence of GUS is decreased by assaying the activity of variants identified in gnomAD, it is still important to estimate the frequency at which mutations absent from gnomAD will be observed in an NBS scenario. In this situation, we assume that molecular screening is carried out in newborns who are flagged as having abnormal blood sulfatide levels and ARSA activity. Individuals with a GUS (two previously unobserved variants, or a previously unobserved variant paired with one of known significance) would need more detailed follow-up compared with individuals with two variants of known significance.

We simulated the frequency at which new mutations would be observed in molecularly screened individuals by iteratively generating “patients,” or individuals with two pathogenic mutations. This was done by drawing, with replacement, two mutations weighted by their frequency in gnomAD to generate patient genotypes. Variants were weighted by dividing the frequency of each mutation by the sum of the allele frequency of each mutation being considered. The iteration, or patient, at which each additional mutation was first observed was noted. Patients were generated until each pathogenic mutation was observed at least once. The number of patients needed to identify the 1…*n*th =|gnomAD| mutation was averaged by carrying out 10^5^ different simulations. For this simulation, we defined the set of variants as those annotated as severe, moderate, or mild in the “finalized severity” column of Additional file 3: Table S3 using their allele frequency from each subpopulation defined in gnomAD.

As expected, the first mutation was identified when the first patient was screened for each population. Considering allele frequencies from gnomAD overall, on average, it took 1.12 patients to identify the next variant, reflecting the likelihood that the first MLD patient will be heterozygous. As more individuals are screened, novel mutations will be encountered less frequently; on average, one will screen 90 individuals between the 49th and 50th novel mutations, and 591 between the 99th and 100th novel mutations encountered.

This simulation modeled the rate at which known mutations in gnomAD would be observed in an NBS scenario. To extend this trend to variants not included in gnomAD, we used piecewise cubic interpolation to fit a curve to the average number of patients screened to observe 1…*n* =|gnomAD| mutations, then predicted the number of patients screened to observe additional variants. The number of mutations with non-zero allele frequencies in each subpopulation in gnomAD, as well as the number of patients screened before encountering a novel mutation, is shown in Additional file 11: Table S10. We estimated, using overall allele frequencies, that one will have to screen 606 patients before identifying the *n* + 1 = 106th novel mutation not found in gnomAD. This assumes that these alleles are randomly assorted from generation to generation. Values will vary greatly when considering each subpopulation. For example, when using allele frequencies from the Ashkenazi population, only 3.48 patients on average will need to be screened before identifying a mutation not in gnomAD, but there were only 3 mutations with non-zero allele frequencies in gnomAD in this population, and MLD is predicted to be virtually non-existent in this population. A similar trend was observed in the Finnish, African, and Latino populations. Novel variants would be identified after screening a small number of patients, but the incidence of MLD in these populations is expected to be low. This implies that while novel mutations in patients will be common, when patients are actually identified, they would still be encountered only after a very large number of births in the population overall. On the other hand, in the NFE population, 62 variants were considered, and it is estimated to take 411 screened patients on average before a novel mutation is encountered. We expect the incidence of MLD to be the greatest in this population (1 in 83,277). Dividing the rate at which novel alleles would be encountered in MLD patients by the expected incidence, we estimate that novel alleles on average would be encountered only in 1 in 34,226,847 births overall.

These estimates relied on the assumption that the most common pathogenic variant in each of these subpopulations has been identified already, and that the subpopulation is represented in gnomAD by a sufficient number of individuals so as to accurately estimate its allele frequency. Even when assuming random mating, overall allele frequencies still do not reflect the overall proportion of each subpopulation and are heavily biased toward NFEs.

## Discussion

We set out to develop a more comprehensive understanding of genotype–phenotype relationships in MLD patients in order to better interpret sequence data as a part of the process for NBS, as well as post-NBS patient follow-up, for MLD. NBS for MLD patients is of critical importance since the clinical experience to date suggests that patient outcomes are improved only when treatment is initiated prior to the onset of symptoms [1, 5–7].

We present a two-part analysis of genotype–phenotype relationships for MLD, using both data from the patient literature and a high-throughput cellular model for the quantification of ARSA enzymatic activity by liquid chromatography–tandem mass spectrometry (LC–MS/MS). The results of these analyses informed the development of a phenotype matrix; a look-up table that serves as a tool to predict MLD phenotype based on a patient’s *ARSA* alleles. An important feature of the phenotype matrix is that it accounts for a class of variants, which we refer to as “mild,” that may result in a disease phenotype in some instances, for example, when paired with a “moderate” or “severe” mutation, but may have a low probability of causing a disease phenotype when paired with another “mild” mutation. We provide evidence that p.R86Q and p.I181S may be two such “mild” mutations in *ARSA*, and believe this phenomenon is not unique to MLD. For example, in Pompe disease, the most frequent mutation in the *GAA* gene in patients is the c.-32-13 T > G splicing variant [25–27]. This mutation is almost exclusively found in later-onset Pompe disease and is frequently paired with variants known to have little to no residual acid alpha-glucosidase (GAA) activity [25]. To date, only a small number of patients who are homozygous for the c.-32-13 T > G variant have been reported, although, based on an allele frequency of roughly 0.5% in the NFE population, homozygous individuals should occur at an incidence of 1 in 40,000 births and be the most common genotype in Pompe disease [25, 28, 29]. The various presentation of patients with homozygous c.-32-13 T > G variants may be attributed to modifying genes, other *GAA* variants, or environmental factors [28], and therefore long-term follow-up studies are required. Variants in other genes associated with autosomal recessive disease exhibit similar behavior: p.Y201C in *ARSB* (mucopolysaccharidosis (MPS) VI) [30, 31]; p.G269S in *HEXA* (Tay–Sachs disease) [32]; p.R229Q in *NHPS2* (nephrotic syndrome) [33, 34]; p.C759F in *USH2A* (found in the homozygous state in retinitis pigmentosa patients, but not Usher syndrome [35]; and p.A138E in *TMPRSS3* (non-syndromic hearing loss and deafness) [36]. It should be noted that, while we believe there is a low probability that individuals with two “mild” variants associated with an autosomal recessive disease will have a phenotype, it does not rule out the possibility that symptoms are so mild they go unrecognized, or patients are diagnosed with a different disease. Indeed, several cases of adult-onset MLD have initially been characterized as Alzheimer’s disease [37, 38]. Current ACMG guidelines can lead to conflicting interpretations of pathogenicity in ClinVar for “mild” variants, as they can satisfy strong criteria for being benign by having non-segregation with disease (ACMG criteria BS4 for classifying benign variants) in homozygous individuals in spite of frequently being identified in *trans* with a pathogenic variant in patients (ACMG criteria PM3 for classifying pathogenic variants) [14].

Among the results from this study, a few points stand out. First, we observed that 36% of VUS tested in our assay had levels of enzymatic activity that were consistent with potentially being pathogenic. More importantly, 26% of the tested VUS displayed ARSA activity in the 0–3% range and are thus predicted to be severe. This argues that NBS based on first-tier whole genome sequencing would miss a substantial number of MLD patients. This is also apparent from the fact that the incidence of MLD using patient-based severity assignments was estimated to be 1 in 122,395, and increased to 1 in 83,277 (closer to the current estimated value of about 1 in 40,000–100,000) only after inclusion of VUS predicted to be pathogenic by our ARSA activity data [2].-

Second, by testing the set of VUS contained in gnomAD, we generated biochemical data for the vast majority of mutant *ARSA* alleles in the population. In fact, the estimated incidence of GUS (genotypes of unknown significance) was reduced by 98% in almost every subpopulation represented in gnomAD. When taking into account variation not represented in gnomAD, we show that such alleles will be increasingly rare and will be encountered infrequently in a NBS scenario. For example, we estimate that alleles not tested in this publication will be encountered in only 1 in 34,226,847 births in the NFE population. Finally, using patient-based severity of alleles, it was found that the MLD phenotype can be predicted from the genotype with up to 76% accuracy.

However, we also note that our approach has several limitations. First, the effects of other modifiers, both genetic and biochemical, on ARSA activity are not accounted for in both activity-based and patient-based models. For example the well-known *ARSA* pseudo-deficiency allele, c.*96A > G, is reported to reduce *ARSA* activity to 10% of control in vivo [23, 39, 40]. Although pseudo-deficiency variants are not, by definition, pathogenic, c*96A > G is common enough to have been observed in cis with pathogenic variants in MLD patients where it may exacerbate the MLD phenotype in unexpected ways [41, 42]. A similar finding has also been described in Krabbe disease [43]. Such additive genetic effects may also explain some of the discrepancies between the severity predicted by the patient-based severity analysis and clinical observation, and the high entropy seen for certain genotypes. As evidenced by the data on the p.E384K variant, our assay will overlook the impact of variants on splicing when using the CDS construct. We propose a genomic construct that can be utilized to detect splice modulating variants; however, it is only suitable for genes within the packaging capacity of the pUC57 plasmid. RNA sequencing may be another useful and complementary tool in this scenario. Last, although HEK293 is a human cell line, it is not an exact replica of the disease-relevant cells or tissue.

Together, these results suggest genetic and biochemical data complement each other, and we propose both strategies be used in tandem, with the LC–MS/MS-based biomarker and enzymatic assays as the first-tier and second-tier assays for MLD NBS, followed by genetic testing. We demonstrate that combining these methods increases diagnostic yield and minimizes false negatives. For instance, VUS can be characterized with our biochemical assay and previously classified pathogenic variants that exhibit spuriously high activity would be detected through sequencing patients. Additionally, it is hoped that, following further validation, the genotype data within this article could be considered together with biochemical and clinical data from diagnosed patients, to better guide follow-up and treatment strategies individualized toward patients’ needs and expected disease progression. The strategy we present may also be of value to other recessive rare disorders.

## Conclusions

We applied a LC–MS/MS assay of ARSA enzymatic activity to variants curated from multiple sources and observed evidence that 36% of VUS may be pathogenic. We also developed a phenotype matrix that can be used as a tool to help predict the severity of MLD based on a patient’s *ARSA* genotype. When applied to the test set of clinically reported MLD patients, the tool displays an accuracy of 76% accuracy. Our strategy is applicable to other recessive diseases and provides a step forwards in VUS interpretation during NBS. By combining biochemical and genetic information, we show that diagnostic yield can be increased, through the reclassification of false negatives and characterization of VUS, which in turn can support the timely diagnosis of patients. For a heterogeneous progressive disease such as MLD, an understanding of genotype–phenotype relationships and a prompt diagnosis are key to initiating appropriate disease management and planning for the patient’s future needs.

## Methods

### Curation of a patient data set from the literature

Data were collated on all *ARSA* pathogenic variants recorded in ClinVar (https://www.ncbi.nlm.nih.gov/clinvar/) on March 8, 2021, by undertaking the union of all ClinVar annotations with *ARSA* variant information from patient literature [2, 4, 9, 11, 12, 23, 38, 41, 42, 44–83]. ClinVar is a comprehensive archive of human genetic variants and their corresponding interpretations of clinical significance or disease involvement. The data set included 489 cases from 49 articles published between 1991 and 2020. Mutational nomenclature was standardized to *ARSA*’s current Human Genome Variation Society coding (HGVSc) convention, corresponding to reference sequences NM_000487.5 and NP_000478.3 [23]. However, some publications reported genotypes in a mixture of nomenclatures, which index outdated versions of ARSA’s genetic and protein sequences. To standardize each variant reported in the literature, we used TransVar to validate variant coordinates and convert them to match NM_000487.5 and NP_000478.3, if necessary [84]. We also performed a Google Scholar search for “MLD,” “ARSA,” “ASA,” “arylsulfatase A,” and “metachromatic leukodystrophy” on May 6, 2020, to capture further *ARSA* variants. This data set will be referred to as the curated patient data set.

Data on age of onset and severity of MLD were also recorded, based on the literature. If phenotype was not recorded in the literature, patients were categorized into three groups by age of onset: infantile/late-infantile (0 up to 2.5 years), juvenile (2.5 to 16 years), and adult (over 16 years) [1]. We assume that an earlier age of onset is associated with a more severe disease phenotype, with infantile/late-infantile MLD categorized as the most severe. Fifteen duplicates were identified in the literature, and only data from the most recent publication were included for these cases.

Variant frequencies in the curated patient data set were compared with population-wide allele frequencies in gnomAD (version 2.0.1). Clinical significances of variants according to ClinVar were reported using distinct terms (which describe variant pathogenicity with varying degrees of certainty), and all ClinVar annotations referenced in this study are as previously defined [14]. While ClinVar annotates variants according to their pathogenicity, it does not provide information on how severely each mutation may impact the function of the gene in which they occur. Data from the curated patient data set and from our mass spectrometry-based assay were used to assign patient-based severity and activity-based severity to variants respectively.

### Construction of *ARSA* variants for enzyme activity assays

Site-specific mutagenesis of the *ARSA* consensus coding sequence (CCDS Database: 14,100.2) in a pUC57 plasmid was performed by GENEWIZ LLC (South Plainfield, NJ, USA). *ARSA* was under the control of a cytomegalovirus (CMV) promoter, and the control coding sequence, BLA, was reverse-orientated and under the control of an elongation factor 1 (EF1)-alpha promoter. The cDNA construct map is shown in Additional file 4: Fig S4. Mutations were also made in the same plasmid construct containing the genomic *ARSA* transcript (ENST00000216124.5). All constructs were verified via DNA sequencing.

### CRISPR/Cas9 knockout of *ARSA* in HEK293T cells

Synthego (Redwood City, CA, USA) generated a monoclonal *ARSA* knockout cell line, disrupting ENSG00000100299 in HEK293T cells using guide RNA with the sequence CGGCCGGCTCCCGGTTCGGA. Cells were cultured at 37 °C, with 5% CO_2_, in high-glucose Dulbecco’s modified Eagle’s medium (DMEM) (Invitrogen, Waltham, MA, USA) supplemented with 10% (v/v) fetal bovine serum (FBS) (VWR, Radnor, PA, USA), 1X Pen-Strep (Gibco, Waltham, MA, USA), and 1X GlutaMAX-I™ (Gibco). Genomic analysis of *ARSA* sequences from HEK293T clones using the Inference of CRISPR Edits algorithm (ICE, v2.0) identified a homozygous, four-base-pair deletion in exon 2 (Additional file 4: Fig S1). Knockout of endogenous ARSA activity was confirmed by the mass spectrometry assay detailed in the next section. The cell line was authenticated and free of contamination.

### Transfection of *ARSA* variants and enzyme activity assay

Transfections were performed in triplicate (three wells) into HEK293T *ARSA* knockout cells, using a 96-well Shuttle Nucleofector (Lonza, Basel, Switzerland) with a Microlab STAR liquid-handling robot (Hamilton Robotics, Reno, NV, USA). Briefly, 500 ng of plasmid per 3 × 10^5^ cells was transfected using Amaxa solution SF (Lonza) and program CA-138. Three days after transfection, cells were washed twice with 200 µL 0.9% saline and flash frozen at − 80 °C. Cells were harvested by adding 60 µL lysis buffer (20 mM Tris–HCl, 2.5 g/L CHAPS, pH 7.5 ± 0.02) followed by agitation on an orbital shaker for 15 min at room temperature. The plate was centrifuged at 3000* g* for 15 min, and the supernatant was collected for bicinchoninic acid (BCA) total protein (Thermo Fisher Scientific, Waltham, MA, USA), BLA, and ARSA activity assays.

BLA activity was determined by measuring hydrolysis of d_7_-penicillin G (Toronto Research Chemicals, Toronto, ON, Canada) to d_7_-5R,6R-benzylpenicilloic acid. The BLA assay cocktail consisted of 200 µM d_7_-penicillin G substrate and 5 µM 5R,6R-benzylpenicilloic acid internal standard in BLA assay buffer (50 mM Tris–HCl, pH 7.5 ± 0.02). Cell lysate supernatant (10 µL) was combined with BLA assay cocktail (30 µL). The plate was sealed, centrifuged at 3000* g* for 1 min, and agitated at 37 °C for 1 h. The reaction was quenched with 150 µL acetonitrile, and the plate was centrifuged at 3000* g* for 5 min. Supernatant (75 µL) was combined with 75 µL water, and the amount of enzyme product (d_7_-5R,6R-benzylpenicilloic acid) generated during the reaction was quantified using the internal standard, 5R,6R-benzylpenicilloic acid. LC–MS/MS analysis was carried out on a Xevo TQ mass spectrometer coupled to an Acquity ultra-performance liquid chromatography system (Waters Corporation, Milford, MA, USA). Separation of the enzyme product and substrate was achieved using an ACQUITY HSS T3 column (1.8 µm, 2.1 × 50 mm, Waters Corporation) connected to an ACQUITY HSS T3 VanGuard pre-column (1.8 µm, 2.1 × 5 mm, Waters Corporation) at 40 °C. Mobile phase A was water with 0.1% formic acid, and mobile phase B was acetonitrile with 0.1% formic acid. The weak needle wash was 90:10 (v:v) water:acetonitrile with 0.1% formic acid, and the strong needle wash was acetonitrile with 0.1% formic acid. The flow rate was 0.8 mL/min, and the linear gradients were as follows: 0–0.2 min, 10% solvent B; 0.2–1.2 min, 10–45% solvent B; 1.2–1.4 min, 45–100% solvent B; 1.4–1.7 min, 100% solvent B; 1.7–1.71 min, 100–10% solvent B, 1.71–2.0 min, 10% solvent B. 5R,6R-benzylpenicilloic acid and d_7_-penicillin G were eluted at 0.98 and 1.32 min, respectively. The enzyme product and internal standard were detected by multiple reaction monitoring using the following transitions: 360.4 > 160.0 and 353.3 > 160.0. The cone voltage and collision energy were 20 and 15 V, respectively. BLA activity in cell lysate (nmol/h/mg protein) was calculated by multiplying the ion ratio of BLA product to BLA internal standard (blank subtracted) by the nanomoles of internal standard added to the assay (nmol), then dividing by the incubation time (h) and the amount of protein (mg).

ARSA activity assays and activity calculations were performed as previously described [4]. Normalized ARSA activity in cell lysate was calculated by dividing the ion ratio of ARSA product to ARSA internal standard (blank subtracted) by the ion ratio of BLA product to BLA internal standard (blank subtracted). Data from activity assays were used to assign activity-based severity to variants. Variants with known severities and functional impacts from literature were used as controls on each plate (benign variant p.P220L, mild variant p.R290H, and the wild-type construct) to ensure assay consistency across batches.

### Derivation of the phenotype matrix and variant severity definitions

Data from curated patients, gnomAD, and ClinVar data were combined with enzyme activity data to generate a phenotype matrix and define a ruleset for classifying variants by severity. In this process (described in the “Results” section) *ARSA* variants were classified as severe, moderate, or mild according to the phenotype they were associated with. This model enforces that disease severity is a function of residual ARSA activity, and in turn, that residual ARSA activity is a function of the activity of each individual *ARSA* allele.

### Estimation of genotype and phenotype incidence and confidence intervals

In order to model MLD, we defined three sets of variants: severe, moderate, and mild. For each set of variants, we estimated the combined probability ($\widehat{\mathrm{p}}$) and variance (Var) within our data set, using a modification of previously described methods [22]. While it is possible that variants co-occur in any possible combination, we simplified the calculation of the combined frequency of alleles in the population by assuming that variants do not co-occur on the same copy of *ARSA* and sum their frequencies. For example, using the “moderate” category as an example we calculated the overall frequency of this category of variants as:

$$\widehat{\mathrm{p}}\left[Moderate\right]= \sum_{v\in Moderate}\widehat{\mathrm{p}}\left[v\right]$$

Similarly, the variance in the frequency of this set of variants is represented as the sum of each individual variant’s variance:

$$Var\left[Moderate\right]= \sum_{v\in Moderate}Var\left[v\right]$$

Given the phenotype matrix, we calculated the expected incidence and variance of each phenotype.

Again, under our model, we assumed one variant per allele (i.e., mutations were not allowed to co-occur in *cis*). The infantile/late-infantile phenotype, for example, results from patients that possess one severe mutation on each allele. The incidence of infantile MLD was therefore calculated as:

$$E\left[Infantile\right]=\widehat{\mathrm{p}}{\left[Severe\right]}^{2}$$

The variance in infantile/late-infantile incidence was calculated as:

$$Var\left[Infantile\right]=2\left({\left(Var\left[Severe\right]+ \widehat{\mathrm{p}}{\left[Severe\right]}^{2}\right)}^{2}- \widehat{\mathrm{p}}{\left[Severe\right]}^{4}\right)$$

The juvenile phenotype is the result of a severe mutation appearing in the *trans* compound heterozygous state with a moderate mutation. The expected incidence of the juvenile phenotype was calculated as:

$$E\left[Juvenile\right]=2\widehat{\mathrm{p}}\left[Severe\right]\widehat{\mathrm{p}}\left[Moderate\right]$$

The variance was:

$$Var\left[Juvenile\right]=4\left(\left(\left(Var\left[Moderate\right]+\widehat{\mathrm{p}}{\left[Moderate\right]}^{2}\right) \times \left(\widehat{\mathrm{p}}{\left[Severe\right]}^{2}\right)\right)- \left(\widehat{\mathrm{p}}{\left[Moderate\right]}^{2}\times \widehat{\mathrm{p}}{\left[Severe\right]}^{2}\right)\right)$$

The adult form of MLD occurs as the result of having a moderate mutation in each of the two copies of *ARSA* or when a mild and a severe mutation occur in *trans*. Its estimated incidence was calculated as:

$$E\left[Adult\right]= \widehat{\mathrm{p}}{\left[Moderate\right]}^{2}+2\widehat{\mathrm{p}}\left[Severe\right]\widehat{\mathrm{p}}\left[Mild\right]$$

The variance in the expected adult incidence rate was then calculated as the sum of the variance in the expected incidence of individuals with two moderate mutations and those compounds heterozygous for a severe and a mild mutation:

$$Var\left[Adult\right]=Var\left[Moderate, Moderate\right]+Var\left[Severe,Mild\right]$$

where *Var*[*Moderate*, *Moderate*] was calculated as:

$$Var\left[Moderate,Moderate\right]=2\left({\left(Var\left[Moderate\right]+ \widehat{\mathrm{p}}{\left[Moderate\right]}^{2}\right)}^{2}\right)- \widehat{\mathrm{p}}{\left[Moderate\right]}^{4}$$

and *Var*[*Severe*, *Mild*] was calculated as:

$$Var\left[Severe,Mild\right]=4\left(\left(\left(Var\left[Severe\right]+ \widehat{\mathrm{p}}{\left[Severe\right]}^{2}\right) \times \left(Var\left[Mild\right]+ \widehat{\mathrm{p}}{\left[Mild\right]}^{2}\right)\right)- \left(\widehat{\mathrm{p}}{\left[Severe\right]}^{2} \times \widehat{\mathrm{p}}{\left[Mild\right]}^{2}\right)\right)$$

Although the variance in the infantile/late-infantile and juvenile incidence estimates could be added, there are mutations that contribute to the adult phenotype and both the infantile/late-infantile and the juvenile phenotype. Because of this, these calculations were not independent. It was therefore not possible to obtain the overall variance in disease incidence by simply summing the variance of each phenotype.

Calculating the overall variance in incidence from gnomAD was simplified by first calculating the variance in the expected incidence of individuals who are compound heterozygous for a severe and a moderate mutation:

$$Var\left[Severe,Moderate\right]=4\left(\left(\left(Var\left[Severe\right]+ \widehat{\mathrm{p}}{\left[Severe\right]}^{2}\right) \times \left(Var\left[Moderate\right]+ \widehat{\mathrm{p}}{\left[Moderate\right]}^{2}\right)\right)- \left(\widehat{\mathrm{p}}{\left[Severe\right]}^{2} \times \widehat{\mathrm{p}}{\left[Moderate\right]}^{2}\right)\right)$$

The variance in the expected incidence of individuals who are compound heterozygous for a severe and a moderate or mild mutation could then be calculated as:

$$Var\left[Severe\left(Mild+Moderate\right)\right]=4 \left(Var\left[Severe, Mild\right]+Var\left[Severe,Moderate\right]+2E\left[Moderate\right]E\left[Mild\right]Var\left[Severe\right]\right)$$

The variance in the infantile/late-infantile phenotype and the moderate/moderate phenotype could then be added, along with twice their respective covariance values:

$$Var\left[Overall\right]=Var\left[Severe\left(Mild,Moderate\right)\right]+Var\left[Infantile\right]+Var\left[Moderate/Moderate\right]+2Cov\left[Severe\left(Mild+Moderate\right),Infantile\right]+2Cov\left[Severe\left(Mild+Moderate\right),Moderate/Moderate\right]$$

With each covariance calculated as:

$$Cov\left[Severe\left(Mild+Moderate\right),Infantile\right]=2E\left[Severe\right]Var\left[Severe\right]2E\left[Mild+Moderate\right]$$

and

$$Cov\left[Severe\left(Mild+Moderate\right),Moderate/Moderate\right]=2E\left[Moderate\right]Var\left[Moderate\right]2E\left[Severe\right]$$

Leveraging the equations above and variant allele frequencies across multiple populations, the incidence of each MLD subtype was calculated from the patient-based severity, activity-based severity, and finalized assay severity assignments within Table 4 and Additional file 10: Table S9. Ninety-five percent confidence intervals (CIs) are provided for the incidence of each form of MLD, as previously described [22].

## Supplementary Information

**Additional file 1: Table S1.** Curated patient data set. Variants, age of onset, and severity phenotypes are shown for MLD patients curated from the literature. Nomenclature for *ARSA* variants has been updated, and for papers using the previous annotation system a +2 amino acid or +6 nucleotide coordinate shift has been applied. Cases for which this coordinate shift has been used are labeled in the Comments column. ARSA, arylsulfatase A; HGVSc, Human Genome Variation Society coding variant; HGVSp, Human Genome Variation Society protein variant.

**Additional file 2: Table S2.** Tabulated genotypes. Consolidation and summary of all genotypes from curated MLD patients. For each genotype, the breakdown of all associated phenotypes in the literature is provided. Entropy, a measure of phenotypic variability for a genotype, and the phenotype matrix’s accuracy in predicting phenotypes are also included. ARSA, arylsulfatase A; CDS, coding sequence.

**Additional file 3: Table S3.** Comprehensive variants. Summary of all variants present in the curated patient data set, all variants from gnomAD, and novel variants reported by MLD Foundation or NBS. All columns are described in the table legend. CDS, coding sequence; GRCh37, Genome Reference Consortium Human Build 37; SNP, single nucleotide polymorphism; WT, wild-type.

**Additional file 4: Fig S1.** Disruption of *ARSA *by CRISPR/Cas9. ICE data showing a four-base-pair deletion in exon 2 of *ARSA *produced by HEK293T cells. ARSA, arylsulfatase A; CRISPR, clustered regularly interspaced short palindromic repeats; ICE, Inference of CRISPR edits. **Fig S2.** Correlation of enzyme activity severities and severities predicted by *in silico *methods. SIFT, PolyPhenand REVELscores for *ARSA* variants plotted as a function of the percentage of wild-type enzyme activity of *ARSA* variants expressed in HEK293T cells. WT, wild-type. **Fig S3.** Results of numeric simulation and analytically derived confidence intervals for the overall incidence of MLD using “All” allele frequencies in gnomAD. Blue bars represent a histogram of observed incidence rates from numeric simulation. Dashed red lines represent the upper and lower empirical 95% confidence intervals for the distribution generated by numeric simulation. The black dashed line represents the mean of the distribution generated by numeric simulation. The grey curve represents the beta approximation of the binomial distribution calculated using the equations for variance described in Methods. Red solid lines represent the analytically defined 95% confidence intervals. The black solid line represents the expected incidence rate”. **Fig S4.** cDNA construct map of plasmid pUC57-KAN.blaM.EIF.CMV.ARSA.bGH used for site-directed mutagenesis of *ARSA*. Expression of *ARSA* by the pUC57 plasmid is driven by the CMV promoter with expression of reverse-oriented beta-lactamase driven by an EF1-alpha promoter. ARSA, arylsulfatase A; bGH, bovine growth hormone; bp, base pair; CMV cytomegalovirus; EF1, elongation factor 1; EIF, eukaryotic initiation factor; SV40 simian virus 40.

**Additional file 5: Table S4.** Enzyme activity of *ARSA *variants in a CDS construct. Summary of all data used to calculate mean CDS ARSA activity. Variants with negative mean percentage of wild-type activity exhibited lower levels of sulfatide catabolism than the plate blank. ARSA, arylsulfatase A; BCA, bicinchoninic acid; BLA, beta-lactamase; CDS, coding sequence; HGVSc, Human Genome Variation Society coding variant; HGVSp, Human Genome Variation Society protein variant; WT, wild-type.

**Additional file 6: Table S5.** Enzyme activity of ARSA variants in a genomic construct. Summary of all data used to calculate mean genomic ARSA variant activity. Variants with negative mean percentage of wild-type activity had less sulfatide catabolism than the plate blank. While multiple VUS in gnomAD and the literature are implicated as splice variants, testing all mutants was cost-prohibitive. We triaged variants for testing using the criteria listed in ‘Rationale for testing’. These results provide proof-of-concept for screening in genomic constructs and highlight the technique’s utility in identifying cryptic splice variants. ARSA, arylsulfatase A; BCA, bicinchoninic acid; BLA, beta-lactamase; CDS, coding sequence; gnomAD, Genome Aggregation Database; HGVSc, Human Genome Variation Society coding variant; HGVSp, Human Genome Variation Society protein variant; VUS, variant of unknown significance; WT, wild-type.

**Additional file 7: Table S6.** Percent reduction of genotypes of unknown significance (GUS) with activity-based severity data. Burdens for each MLD subtype were predicted using allele frequencies from gnomAD and our phenotype matrix. Frequencies of unknown genotypes are reported across various populations. Calculations were performed using patient-based severity, as defined by our variant-severity criteria, and repeated using activity-based severity determined by MS/MS. Both one-in incidences and allele frequencies are provided, and the percent reduction in GUS when activity-based severity data are used instead of patient-based severity is shown. Classifying VUS by activity-based severity greatly reduces the number of genotypes with indeterminant phenotypes. gnomAD, Genome Aggregation Database; GUS, genotypes of unknown significance; MS/MS, tandem mass spectrometry; NFE, non-Finnish European; VUS, variants of unknown significance.

**Additional file 8: Table S7.** ClinVar and variant severity. Tabulation of ClinVar annotations and associated patient-based severity and activity-based severity assignments.

**Additional file 9: Table S8.** Predicted individual genotype incidence rates in all populations. The expected incidence of an individual genotype was calculated as the product of the allele frequency of the two mutations comprising the genotype for each subpopulation reported in gnomAD. ARSA, arylsulfatase A, CDS, coding sequence

**Additional file 10: Table S9.** Analytical analysis of disease burden. Burdens for each MLD subtype were predicted using allele frequencies from gnomAD and our phenotype matrix. Calculations were performed using patient-based severity, as defined by our variant-severity criteria, and repeated using activity-based severity determined by MS/MS. 95% percent confidence intervals were calculated using the analytical method, except for the “Unknown” and “Asymptomatic” incidence rates, which rely on 95% confidence intervals calculated through numeric simulation. gnomAD, Genome Aggregation Database; MS/MS, tandem mass spectrometry.

**Additional file 11: Table S10.** Number of mutations with non-zero allele frequencies in each subpopulation in gnomAD. Number of patients screened before encountering a novel mutation are also shown by subpopulation. gnomAD, Genome Aggregation Database.

**Additional file 12.** Review history.

## Data Availability

All data generated and analyzed during the study are available in this published article and supplementary tables. Curated patient data are available in Additional file 1: Table S1. Source articles for curated patients are listed in column F of this table. Curated individual variants are available in Additional file 3: Table S3. Allele frequencies, allele counts, and counts of homozygous individuals of these variants from gnomAD, when available, are included in columns T through BJ [13]. ClinVar annotations as recorded in ClinVar (https://www.ncbi.nlm.nih.gov/clinvar/) on March 8, 2021, are recorded in column H.
