# Supplementary figures and images for: Predicting disease severity in metachromatic leukodystrophy using protein activity and a patient phenotype matrix
Source: Genome Biol. 2023 Jul 21;24:172. doi: 10.1186/s13059-023-03001-z (PMC10360315; doi:10.1186/s13059-023-03001-z)

Fig S1

Relative contribution of each sequence (normalized)

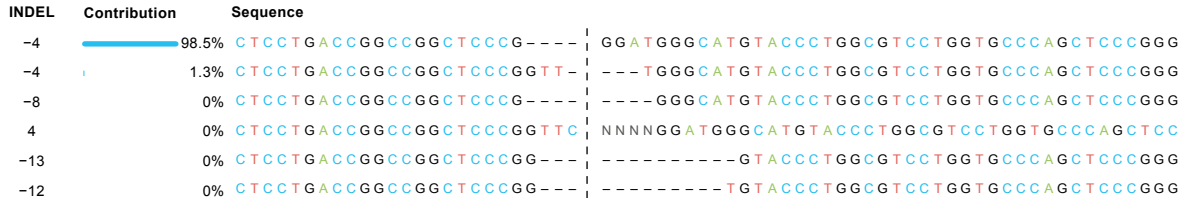

Fig S2

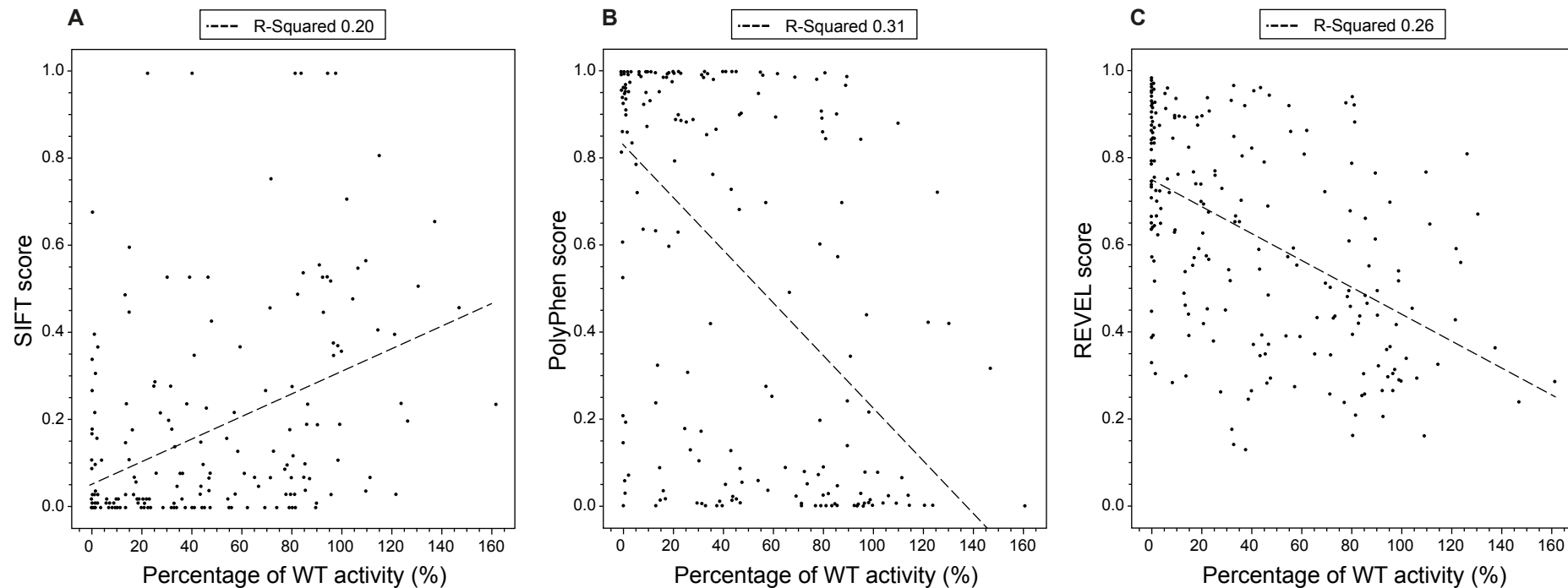

Fig S3

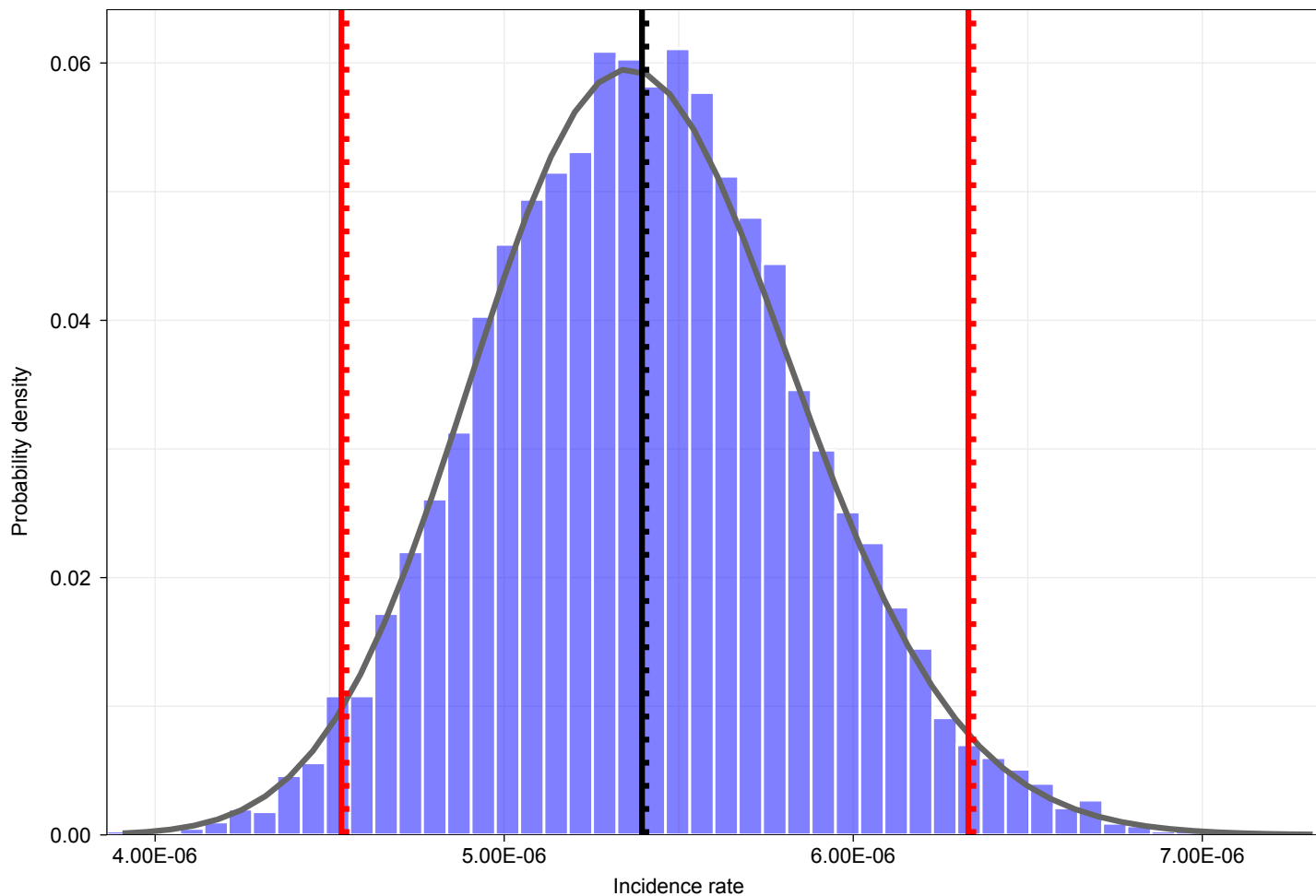

Fig S4

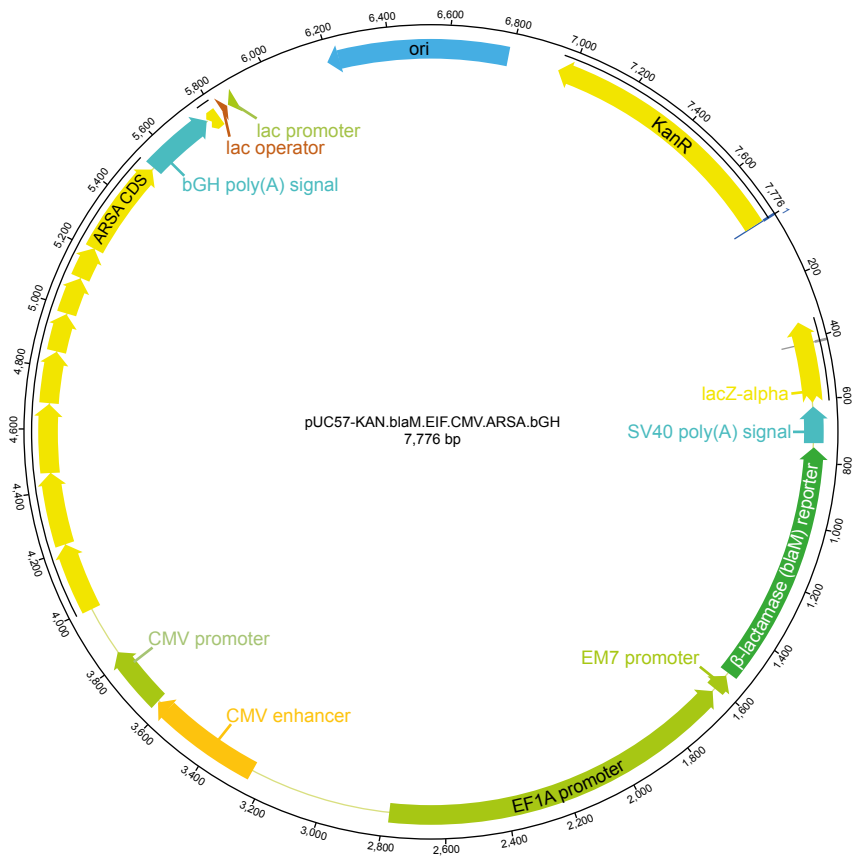

Supplement: Supplementary file 4 — Additional file 4: Fig S1. Disruption of ARSA by CRISPR/Cas9. ICE data showing a four-base-pair deletion in exon 2 of ARSA produced by HEK293T cells. ARSA, arylsulfatase A; CRISPR, clustered regularly interspaced short palindromic repeats; ICE, Inference of CRISPR edits. Fig S2. Correlation of enzyme activity severities and severities predicted by in silico methods. SIFT, PolyPhenand REVELscores for ARSA variants plotted as a function of the percentage of wild-type enzyme activity of ARSA variants expressed in HEK293T cells. WT, wild-type. Fig S3. Results of numeric simulation and analytically derived confidence intervals for the overall incidence of MLD using “All” allele frequencies in gnomAD. Blue bars represent a histogram of observed incidence rates from numeric simulation. Dashed red lines represent the upper and lower empirical 95% confidence intervals for the distribution generated by numeric simulation. The black dashed line represents the mean of the distribution generated by numeric simulation. The grey curve represents the beta approximation of the binomial distribution calculated using the equations for variance described in Methods. Red solid lines represent the analytically defined 95% confidence intervals. The black solid line represents the expected incidence rate”. Fig S4. cDNA construct map of plasmid pUC57-KAN.blaM.EIF.CMV.ARSA.bGH used for site-directed mutagenesis of ARSA. Expression of ARSA by the pUC57 plasmid is driven by the CMV promoter with expression of reverse-oriented beta-lactamase driven by an EF1-alpha promoter. ARSA, arylsulfatase A; bGH, bovine growth hormone; bp, base pair; CMV cytomegalovirus; EF1, elongation factor 1; EIF, eukaryotic initiation factor; SV40 simian virus 40. [file 13059_2023_3001_MOESM4_ESM.pdf]
