# Supplementary material for: Predicting disease severity in metachromatic leukodystrophy using protein activity and a patient phenotype matrix
Source: Genome Biol. 2023 Jul 21;24:172. doi: 10.1186/s13059-023-03001-z (PMC10360315; doi:10.1186/s13059-023-03001-z)
Supplement: Supplementary file 12 — Additional file 12. Review history. [file 13059_2023_3001_MOESM12_ESM.docx]

Review History

**First round of review**

**Reviewer 1**

**Were you able to assess all statistics in the manuscript, including the appropriateness of statistical tests used?** Yes, and I have assessed the statistics in my report.

**Comments to author:**

This manuscript describes a function analysis of ARSA variants. Results significantly reduce the number of variants of uncertain significance. Below are some comments.

Additional file 3 should contain #CHROM-POS-REF-ALT for ALL variants to help readers to use the data. The genomic build number, GRCh37 or GRh38, should also be stated in the title line.

Page 8 line 175 "near the C-terminus of the protein" needs to be more specific. Was it defined as a percentage of other protein sequence, or the number of basepairs, or the last exon?

What is the mechanism of not defining all frameshift variants as loss of function, but only those "resulting in stop-gained"?

What is the MaxENT score threshold to define "hits from the splice prediction algorithm MaxENT" on page 11 line 268?

Why the "mean percent variant activity" can be negative?

The authors used p.I181S and p.T393S as an example to show the method of setting ABS thresholds. But the Additional file 5 shows that a Moderate variant has a Mean percent WT activity as high as 70%. How does that fit into the conclusion that "moderate limits were set at 2% to <4% of wild-type activity" on page 12 line 296? Also, with only 2 mild and 2 benign variants that have both ABS and PBS data, the boundaries between moderate-and-mild and mild-and-benign are not going to be accurate.

the acronyms GUS and CDS are never defined.

The authors assessed in silico predictors PolyPhen and SIFT. But those two are old methods. ClinGen supports the usage of meta predictors. The authors should evaluate the most up-to-date methods like those listed in https://doi.org/10.1101/2022.03.17.484479

It is not clear to me how the authors evaluated the accuracy of PBS and ABS on page 17. Did the author used different set of variants for method creation and method evaluation separately? If not, there will be circularity that will inflate the accuracy estimation.

The accuracy, 78% for PBS and 70% for ABS, is low for clinical usage. Therefore, functional assays should be integrated into the ACMG scheme for a more accurate classification, where multiple lines of evidence can be combined. This paper did not try to do so, while there're already many other papers describing such methods for other genes. My suggestion is to change the rule-based ABS thresholds into likelihood ratios for each bin of percentage of WT activity, then the authors can combine multiple lines of evidence by the multifactorial approach. That will also solve the inconsistency issues in the rule-based ABS thresholds, such as that demonstrated by the moderate variant with 70% WT activity.

**Reviewer 2**

**Were you able to assess all statistics in the manuscript, including the appropriateness of statistical tests used?** No, I do not feel adequately qualified to assess the statistics.

**Comments to author:**

This manuscript correlates genotype with phenotype in MLD based on patient presentation and aims to address the challenges of variants of unknown significance by considering both the observed clinical phenotype and ARSA enzyme activity expressed in a laboratory based in vitro cell model.

The manuscript has a comprehensive assessment of variants in the ARSA gene and will provide a valuable resource to clinicians and scientists working within the field of MLD. The study has involved a large body of work and includes an extensive record of variants in the ARSA gene in the supplementary information. I recommend that the manuscript is accepted for publication. I have some minor comments for consideration:

1. Manuscript line 177 'any mutation not found in trans with a severe mutation'. Should this not read 'any mutation found in trans with a severe mutation'?

2. The authors have proposed a phenotype matrix that suggests mild variants will only be clinically significant when in trans with a severe variant. Patients with mild variants may not present until late middle age with an adult neurological phenotype that is not identified as MLD, as metabolic investigations are not always considered for this patient group. While this would not impact on potential patient treatment options following NBS, without clear evidence that mild variants give an asymptomatic wild-type phenotype it would be more appropriate to state 'likely asymptomatic' in the matrix. The manuscript would benefit from consideration of this issue.

3. Abbreviations: the manuscript is complex and would benefit from a clear guide to the abbreviations used to assist the expected wide readership.

4. This paper demonstrates a relationship between residual enzyme activity and clinical phenotype in the cell-based model. This would be a challenging diagnostic strategy outside of a research setting. Is there any information as to whether this relationship is reflected in easily available patient samples?

5. In the discussion the authors focus on the role of NBS for MLD based on genetic analysis as the primary screening test. The manuscript would benefit from discussion on the role of the data in support of a NBS program where the strategy is to use sulphatide followed by ARSA activity. There is a wealth of information in this document that can potentially help with clinical phenotype predication, this could be discussed further.

These recommendations will not require significant alterations to the manuscript and should be easily addressed by the authors.

**Reviewer 3**

**Were you able to assess all statistics in the manuscript, including the appropriateness of statistical tests used?** No, I do not feel adequately qualified to assess the statistics.

**Comments to author:**

Thank you for the opportunity to review the manuscript titled "Predicting disease severity in metachromatic leukodystropy using protein activity and a patient phenotype" submitted by Trinidad et al. The patient phenotype matrix created aimed to predict disease severity in MLD is interesting and novel for NBS and healthcare professionals, and the long-term follow-up plan; post NBS put forth based on this matrix is interesting.

Although this work represents an interesting and an advanced concept of phenotyping patients for LSDs based on the variants detected and in particular MLD, however at times it is hard to understand the purpose of creating this matrix and how enzyme activity data and variant data could correlate accurately and clearly with patient phenotype presentations based on this matrix.

I am sure that this manuscript idea and concept, and suggested algorithm is going to be of interest to LSD community at large, especially in the arena of new born screening for LSDs and MLD specifically.

Major concerns:

1. Too many abbreviations used throughout the manuscript without a clearly spelling out of the abbreviations; need to be spelled out the firsttime abbreviation is used (examples: NFE, CDS, PBS, GUS etc.).

2. Some statements are not making sense - like line 149 "the most common alleles in patients are the most common alleles in the general population"

3. Authors have created an extensive table (2) showing burden of disease in various World populations, essentially population frequencies of MLD- but they have failed to show a simple table of common variants detected in MLD with measured enzyme activity levels associated with these variants. This simple table would add a big value to the readers of this paper.

4. Severity rules created by the authors - what clinical symptoms are these rules based on, there is no clear mention of the phenotypic correlations.

5. Result section seems exhaustive and redundant. The phenotypic categorization of patients is not clear, no clinical symptoms described or classification based on clinical criteria are listed at all. This section can be tightened and clarified for the readers.

6. Authors have done functional analysis on 281 ARSA variants using HEK293T cell lines, it would be nice to show how many control variants authors had used to validate their assays (As pee ACMG functional analysis guidelines) and what was the percentage enzyme activity measured for each variant in their assay. This detailed table would be very helpful in classification of these VUS as Likely pathogenic/Pathogenic alleles in Clingen/Clinvar variant assigments and very helpful for MLD NBS and genetic community at large. Following clear ACMG guidelines of variant classification is very important.

7. There is no mention of reagents used of MS/MS enzyme activity measurements, Method validations and method limitations for enzyme activity measurement using MS/MS methodology.

What is BLA? stability of reagents etc. is missing.

8. Authors' suggestion to rely in ARSA gene sequencing as a primary screening method for MLD NBS is not convincing. It is clearly costly and clearly not diagnostic at this point. They are stating at the end that enzyme activity assay may be more definitive for diagnosis of MLD in NBS setting so when and where is the use of their phenotypic matrix that they have created in MLD NBS arena.

9. Results and Methods can be worked on to make it precise and clear for the readers. Both these sections are too long and exhaustive for the reader and still missing the essential information reader is looking for - variants identified with enzymatic data for each variant.

**Authors Response**

**Point-by-point responses to the reviewers’ comments:**

**Reviewer #1**:

1. Additional file 3 should contain #CHROM-POS-REF-ALT for ALL variants to help readers to use the data. The genomic build number, GRCh37 or GRh38, should also be stated in the title line.

**We used GRCh37 and added #CHROM-POS-REF-ALT to column HGVSg_GRCh37 (column C), where possible. Twelve variants remained for which we could not determine the HGVSg from either the HGVSc or other sources. We believe the benefit of including these variants outweighs the cost of removing them.**

2. Page 8 line 175 "near the C-terminus of the protein" needs to be more specific. Was it defined as a percentage of other protein sequence, or the number of basepairs, or the last exon?

**We have updated our language to be more precise, and replaced “near the C-terminus of the protein” with “terminal,” and have referenced [14], which showed that a smaller fraction of terminal-truncating mutations and inframe insertions/deletions exhibited population genetic signatures indicating an impact on gene function when compared with frameshift or stop gained mutations (lines 198–202).**

3. What is the mechanism of not defining all frameshift variants as loss of function, but only those "resulting in stop-gained"?

**We apologize for the imprecise language. We meant to allow for in-frame insertions or deletions that may not impact protein function. As stated above, we have added a supporting reference for this, and have changed the language in the manuscript (lines 198–202) to read “Loss of function (LoF) mutations (stop-gained or out-of-frame insertions/deletions leading to a stop-gained mutation) were classified as severe since these almost universally eradicate ARSA enzyme activity (an exception might be a terminal, stop-gained codon) [14].**

4. What is the MaxENT score threshold to define "hits from the splice prediction algorithm MaxENT" on page 11 line 268?

**Due to constraints, we selected a subset of variants (n=8) that MaxENT classified as gain-of-splice-acceptor, with a greater than 40% difference in the wild-type and mutant scores. These criteria were added to the “Selection of *ARSA* variants for activity assays” section (lines 308–310).**

5. Why the "mean percent variant activity" can be negative?

**All enzyme activities are reported as the measured activity minus that seen in the blank in which cell lysis buffer only was added. Since the blank is finite, the enzymatic activity can be negative when it is very close to zero and due to experimental error is observed to be just below that measured in the blank. Negative values were close to zero, but were reported as measured for precision and transparency. However, for the purpose of genotype–phenotype analysis, negative values reflect null enzymatic activity. We clarified in the manuscript body, by adding the following to lines 332–336: “For precision, enzymatic activity is reported as directly calculated and may include negative values, due to the nature of blank subtractions. Variant activity can be negative when the enzymatic activity is close to zero and gives an observed value slightly lower than observed with the blank in which only cell lysis buffer was used. For the purpose of genotype–phenotype analysis, negative values reflect null enzymatic activity.”**

6. The authors used p.I181S and p.T393S as an example to show the method of setting ABS thresholds. But the Additional file 5 shows that a Moderate variant has a Mean percent WT activity as high as 70%. How does that fit into the conclusion that "moderate limits were set at 2% to <4% of wild-type activity" on page 12 line 296? Also, with only 2 mild and 2 benign variants that have both ABS and PBS data, the boundaries between moderate-and-mild and mild-and-benign are not going to be accurate.

**We agree our assay contains outliers. The text addresses cases where a variant’s activity-based severity is incongruous with its assigned patient-based severity or “Final Severity” in Additional File 3. Some of these outliers may be explained by unforeseen variant effects, such as splicing (e.g. p.E384K is described in literature as moderate but exhibited benign CDS-activity at 70.22%) or introduction of a protease motif (ex. p.P428L is a moderate allele in literature but possessed severe CDS-activity at 0.04%). In light of these infrequent exceptions, we state our cellular model may not capture the unique properties of all variants in lines 364-374 and dedicated the section entitled “Agreement of enzyme activity and patient-based severity” (line 387) to cover this issue. Additionally, due to confounding issues like patient comorbidities and the low number of genotype–phenotype case-studies described in literature, patient-based severity assignments may be imperfect. To safeguard against this, we required alleles to exist in 5 or more individuals from the patient dataset. We acknowledge this threshold is lower than ideal and might contribute to conflicting activity-based severity and patient-based severity, but were unable to increase this threshold due to data scarcity. We hope future works increase the availability of MLD patient data, so we can refine and increase the accuracy of patient-based severity and activity-based severity assignments. Despite this, however, the activity thresholds defining each subtype are representative, given that the average activity for each category of allele falls within our defined thresholds (severe: 0.2562% WT CDS-activity; moderate: 2.8430% WT CDS-activity; mild: 7.3341% WT CDS-activity; and benign: 43.9281% WT CDS-activity) for variants of known significance (those defined in literature and/or with ≥5 patients).**

7. The acronyms GUS and CDS are never defined.

**Thank you, we have corrected this on lines 384 and 329 respectively.**

8. The authors assessed in silico predictors PolyPhen and SIFT. But those two are old methods. ClinGen supports the usage of meta predictors. The authors should evaluate the most up-to-date methods like those listed in https://www.biorxiv.org/content/10.1101/2022.03.17.484479v1

**REVEL Scores were available for 219 variants, and were used to calculate a two-tailed Pearson correlation with 'CDS percent WT activity.' We removed the correlations with the genomic assay, due to an insufficient number of variants with predictions for these tools (section ‘Performance of *in silico* predictors of VUS enzyme activities [line 462]).**

9. It is not clear to me how the authors evaluated the accuracy of PBS and ABS on page 17. Did the author used different set of variants for method creation and method evaluation separately? If not, there will be circularity that will inflate the accuracy estimation.

**We agree this may have inflated our accuracy estimate. To gain an unbiased assessment it would be best to use one set of patients for variant severity determination and another set for measuring the accuracy of the phenotype matrix. In order to do this, we cross-validated a supervised random forest model using genotype information, biochemical assay information, or a combination of both data as features. In order to do this we trained a random forest model as described in the manuscript. (lines 542–588) We found that accuracy values were slightly lower, as expected.**

10. The accuracy, 78% for PBS and 70% for ABS, is low for clinical usage. Therefore, functional assays should be integrated into the ACMG scheme for a more accurate classification, where multiple lines of evidence can be combined. This paper did not try to do so, while there're already many other papers describing such methods for other genes. My suggestion is to change the rule-based ABS thresholds into likelihood ratios for each bin of percentage of WT activity, then the authors can combine multiple lines of evidence by the multifactorial approach. That will also solve the inconsistency issues in the rule-based ABS thresholds, such as that demonstrated by the moderate variant with 70% WT activity.

**We thank the reviewer for the suggestion and have calculated the diagnostic odds ratio in the results section (lines 448–460). We have emphasized that the false negative rate is decreased when genetic and biochemical information are combined.**

**Reviewer #2**:

1.	Manuscript line 177 'any mutation not found in trans with a severe mutation'. Should this not read 'any mutation found in trans with a severe mutation'?

**Thank you for catching this typo. The typo was amended to 'any mutation found in trans with a severe mutation' (on line 203).**

2.	The authors have proposed a phenotype matrix that suggests mild variants will only be clinically significant when in trans with a severe variant. Patients with mild variants may not present until late middle age with an adult neurological phenotype that is not identified as MLD, as metabolic investigations are not always considered for this patient group. While this would not impact on potential patient treatment options following NBS, without clear evidence that mild variants give an asymptomatic wild-type phenotype it would be more appropriate to state 'likely asymptomatic' in the matrix. The manuscript would benefit from consideration of this issue.

**The reviewer makes a good observation. Mild *ARSA* variants exhibit greater phenotypic variability and lower penetrance, and their residual activity allows many patients to present symptom-free until middle age and late adulthood. We did not choose to update the phenotype matrix, but better described the nature of two genotypes containing mild variants in the discussion (lines 735–763).**

3.	Abbreviations: the manuscript is complex and would benefit from a clear guide to the abbreviations used to assist the expected wide readership.

**We agreed and we limited the use of abbreviations and added definitions where appropriate at first use of each abbreviation.**

4.	This paper demonstrates a relationship between residual enzyme activity and clinical phenotype in the cell-based model. This would be a challenging diagnostic strategy outside of a research setting. Is there any information as to whether this relationship is reflected in easily available patient samples?

**Hong 2020 (citation 4), the original source of the mass spectrometry assay, describes the use of this assay during newborn screening in a clinical setting. This paper describes multiple variants tested directly from patient leukocyte samples.**

5.	In the discussion the authors focus on the role of NBS for MLD based on genetic analysis as the primary screening test. The manuscript would benefit from discussion on the role of the data in support of a NBS program where the strategy is to use sulphatide followed by ARSA activity. There is a wealth of information in this document that can potentially help with clinical phenotype prediction, this could be discussed further. These recommendations will not require significant alterations to the manuscript and should be easily addressed by the authors.

**To address this suggestion, we extended the final paragraph of the introduction (lines 102–111) and discussion (lines 802–822).**

**Reviewer #3**:

Major concerns:

1. Too many abbreviations used throughout the manuscript without a clearly spelling out of the abbreviations; need to be spelled out the first time abbreviation is used (examples: NFE, CDS, PBS, GUS etc.).

**We welcome this advice and have defined our abbreviations.**

2. Some statements are not making sense - like line 149 "the most common alleles in patients are the most common alleles in the general population"

**Thank you for pointing out these ill-formed sentences. We have read through the manuscript to address this. We have replaced the sentence you referenced with the following: “the most frequent pathogenic alleles in the patient dataset are also the most common pathogenic alleles in the general population, as reported in gnomAD” in lines 166–167.**

3. Authors have created an extensive table (2) showing burden of disease in various World populations, essentially population frequencies of MLD- but they have failed to show a simple table of common variants detected in MLD with measured enzyme activity levels associated with these variants. This simple table would add a big value to the readers of this paper.

**We thank the reviewer for their support.** **We tested all missense variants in gnomAD regardless of their frequencies in subpopulations defined in gnomAD. This data is organized in Additional File 3. We also mentioned gnomAD is biased for Non-Finnish European individuals and how this impacts confidence intervals of our incidence estimates (lines 609–610, and 720–721). We have added additional tables of the most frequently identified genotypes in patients (Table 1, line 1474) and pathogenic variants (Table 2, line 254). We have also added an additional table of the expected incidence of each genotype in each population in gnomAD (Additional table 12).**

4. Severity rules created by the authors - what clinical symptoms are these rules based on, there is no clear mention of the phenotypic correlations.

**Patient phenotype annotations are based on van Rappard 2015 (citation 1), using age of onset for each MLD subtype (infantile/late-infantile, juvenile, and adult). Many clinical symptoms overlap between subtypes and age of onset is the clinically established means of organizing patients by phenotypic severity. This is addressed in lines 66-68, as well as the sections titled “Classifying variants using the curated patient data set: Patient-based variant severity rules” (line 187) and “Development of a phenotype matrix” (line 258).**

5. Result section seems exhaustive and redundant. The phenotypic categorization of patients is not clear, no clinical symptoms described or classification based on clinical criteria are listed at all. This section can be tightened and clarified for the readers.

**Each patient phenotype in our dataset was taken directly from literature, and all patient phenotypes were categorized by physicians and based on age of onset. The goal of this paper was to use patient phenotypes to define variant severities, not redefine clinical criteria for diagnosis of MLD.**

6. Authors have done functional analysis on 281 ARSA variants using HEK293T cell lines, it would be nice to show how many control variants authors had used to validate their assays (As pee ACMG functional analysis guidelines) and what was the percentage enzyme activity measured for each variant in their assay. This detailed table would be very helpful in classification of these VUS as Likely pathogenic/Pathogenic alleles in Clingen/Clinvar variant assignments and very helpful for MLD NBS and genetic community at large. Following clear ACMG guidelines of variant classification is very important.

**We list variants of known severity in Fig 3. We have added “Variants with known severities and functional impacts from literature were used as controls on each plate (benign variant p.P220L, mild variant p.R290H, and the wild-type construct) to ensure assay consistency across batches” to the methods (line 937–939).**

7. There is no mention of reagents used of MS/MS enzyme activity measurements, Method validations and method limitations for enzyme activity measurement using MS/MS methodology.

What is BLA? stability of reagents etc. is missing.

**We appreciate the reviewer’s commitment to fostering reproducibility. The assay and reagents for tandem mass spectrometry are as described in Citation 4 (Hong 2020). The BLA abbreviation is already defined on line 322.**

8. Authors' suggestion to rely in ARSA gene sequencing as a primary screening method for MLD NBS is not convincing. It is clearly costly and clearly not diagnostic at this point. They are stating at the end that enzyme activity assay may be more definitive for diagnosis of MLD in NBS setting so when and where is the use of their phenotypic matrix that they have created in MLD NBS arena.

**We agree this point needs to be better clarified. To remedy this**, **we added a new concluding paragraph to the Discussion section that describes how enzymatic activity increases the diagnostic yield for MLD and decreases the false negative rates, in comparison with NGS (lines 802–822). We also replaced lines 70–71 with “through neuroradiological, biochemical, and/or genetic testing [1, 2], with enzymatic-activity screening being the most common approach. [3, 4]”**

**Once an individual is diagnosed with MLD, then we see the phenotype matrix coming into play when trying to anticipate the course of disease in that subject. Current ACMG guidelines also advise a diagnosis in the presence of biallelic pathogenic variants in a gene associated with autosomal recessive disease, yet guidelines may need to be updated to account for instances where two mild mutations may not lead to disease, or lead to onset of symptoms later in life. Furthermore, such mild mutations cause confusion under current guidelines for variant interpretation as they sometimes cause disease when paired with a severe mutation, but may not when paired with another mild mutation. We point out additional variants in genes that behave this way in the manuscript (lines 740–754).**

9. Results and Methods can be worked on to make it precise and clear for the readers. Both these sections are too long and exhaustive for the reader and still missing the essential information reader is looking for - variants identified with enzymatic data for each variant.

**This is a valid point. Enzymatic activity data are available in Additional File 3. To improve navigability, the first sentence of the “ARSA enzyme activity assay” section was updated to read “Assay results and enzymatic activity for each variant are listed in Additional File 3” in line 314.**

**Second round of review**

**Reviewer 1**

The authors have answered all my previous questions. I am satisfied with the answers.

I have only minor concerns about some newly added text in the manuscript. On page 8 line 182, "p.P220L (with a frequency of 0.028 in the Finnish population in gnomAD) ... were considered to be benign in our analysis based on these high frequencies .." Why consider Finnish? Can the authors justify the usage of a fonder population like Finnish, such as calculating the maximum credible population allele frequency (http://cardiodb.org/allelefrequencyapp/). This may also clarify and justify the phrase "high frequencies" in this sentence. However, I am not sure the above website is suitable. Just for the authors to consider.

There are several "gnomic construct" in the manuscript.

Can the authors presents p-values when comparing AUC of two methods?

**Reviewer 2**

Thank you for considering my reviewer comments.

**Reviewer 3**

Thank you for taking all reviewer's comments and suggested edits into consideration to make your manuscript more meaningful to the readers. It has brought more clarity to the paper and would help readers understand the concept of phenotype linked to matrix and MLD variants.

No additional Major or minor changes are needed.
